# Medicinal plant use practice in four ethnic communities (Gurage, Mareqo, Qebena, and Silti), south central Ethiopia

**DOI:** 10.1186/s13002-020-00377-1

**Published:** 2020-05-24

**Authors:** Alemtshay Teka, Zemede Asfaw, Sebsebe Demissew, Patrick Van Damme

**Affiliations:** 1 Department of Biology, College of Natural and Computational Sciences, Kotebe Metropolitan University, P.O. Box 31248, Addis Ababa, Ethiopia; 2 Department of Plant Biology and Biodiversity Management, College of Natural Sciences, Addis Ababa University, P.O. Box 3434, Addis Ababa, Ethiopia; 3 Laboratory for Tropical and Subtropical Agriculture and Ethnobotany, Department of Plants and Crops, Faculty of Bio-Science Engineering, Ghent University, Coupure links 653, 9000 Gent, Belgium; 4 Faculty of Tropical AgriSciences, Czech University of Life Sciences Prague, Kamycka 129, Prague 6 –, 165 21 Suchdol, Czech Republic

**Keywords:** Culture, Ethnic groups, Gurage, Mareqo, Medicinal plants, Qebena, Silti, Traditional knowledge

## Abstract

**Background:**

Ethnic groups throughout the world have developed their own cultures expressed in the form of customs, taboos, and traditional healthcare systems. Traditional medicine system is one of the widespread cultures known throughout the world which is very much tied to cultural practices of the community or ethnic group. Medicinal plant treasure found in Gurage and Silti zones remained poorly characterized and understood. Therefore, this study was conducted in four ethnic groups: three from Gurage zone (Gurage, Qebena, and Mareqo) and one from Silti zone (Silti) which have lived in close proximity and contact for many centuries in the respective zones. In the present study, unique and shared cultural elements in connection to traditional herbal medicine were examined through investigation of the diversity of medicinal plants. Moreover, attempts have been made to determine similarities among the society in the medicinal plants they have used in general and in medicinal plant species considered culturally most important.

**Methods:**

In a study that involved 320 randomly sampled informants, semi-structured interviews, focus group discussions, and participant observation were used and qualitative and quantitative data were collected. Descriptive statistics, rank order priority (ROP), informant consensus factor, Jaccard similarity coefficient, and clustering were used for data analysis.

**Results:**

A total of 244 medicinal plant species and a fungal species used to treat human and/or livestock ailments were documented. The number of plants (80 plants, 33 %) with ROP value greater than 50% were considerably fewer than that of plants with ROP < 50% (164, 67 %). Jaccard similarity index and clustering analysis for all cited plants, among the respective studied districts, indicated that grouping generally followed the existing ethnic origin. On the contrary, clustering based on culturally important medicinal plant species (80 plant species, score ROP ≥ 50%) showed the influence of proximity and geographical orientation rather than ethnic relation.

**Conclusions:**

Culturally, most important plants (80 spp.) are widely used and best shared with nearby communities and this could imply current (new) knowledge being practiced in the communities. This knowledge must be documented and better utilized in a modern way including modernized use of traditional medicinal plants.

## Introduction

Ethnic groups throughout the world have developed their own cultures expressed in the form of customs, taboos, songs, traditional foods, and healthcare systems. Traditional medicine selected based on several thousands of years of experience has been a major aspect of cultural heritage, and it is widely known throughout the world [1, 2]. Like other kinds of local knowledge, traditional medicine is also very much tied to cultural practices of the community or ethnic group [3, 4]. Its role in the healthcare system is enormous and widely recognized. The word “culture” refers to the characteristics and knowledge of a particular group of people, defined by everything from language, religion, cuisine, social habits, song, story, and arts [5]. In the present study, the type of plants used in a group of people in their traditional health care system is considered as part of culture.

Ethnomedicinal knowledge, which develops from the interaction of a given culture with the local biophysical environment or locally available plants, is diverse, and sometimes, it could be ecosystem and ethnic community specific [6, 7]. Factors such as social, ecological, cultural background (incl. religious, linguistic), and ancestral inheritance determine the kind of traditional herbal knowledge developed in a community. Owing to these facts, herbal knowledge varies hugely across different communities, geographic settings, or ethnic groups [7–9]. The influence of cultural background and ancestral inheritance could be seen in the variation of people’s perceptions and plant use preference in a community inhabiting the same geographical or ecological area, facing similar environmental factors [9, 10]. As a result, comparative studies in traditional knowledge and plant use culture between communities or ethnic groups give an opportunity to investigate how the local flora is understood and used in daily life, health practices, and ultimately for survival under different cultural settings [11].

Several studies conducted in different corner of the world have shown the variation that exists in plant use knowledge and ethnomedicinal healing systems across cultures and agro-ecology [12–16]. Traditional medicinal plants use could also vary among communities within the same ethnic group [17] and geographic area [18]. Thus, medicinal plant use practice can be considered to show the cultural differences that may possibly exist between traditional societies or ethnic groups inhabiting in similar/different geographic locations.

Recently, the knowledge of traditional peoples and accompanied systems is disappearing at increasing rate. Besides medicinal plant treasure found in Gurage and Silti zones is not fully explored. In light of these scenarios, the present study is conducted to document the traditional medicinal plants known in the societies of the two zones. The study area harbor four ethnic groups (viz. Gurage, Qebena, Mareqo, and Silti) which have lived in close proximity and contact for many centuries. In the present study, unique and shared cultural elements in connection to traditional herbal medicine were examined through investigation of the diversity of medicinal plants. Moreover, attempts have been made to determine similarities among the society in the medicinal plants they have used in general and in medicinal plant species considered culturally most important.

## Methods

### Study area

**Fig. 1:**
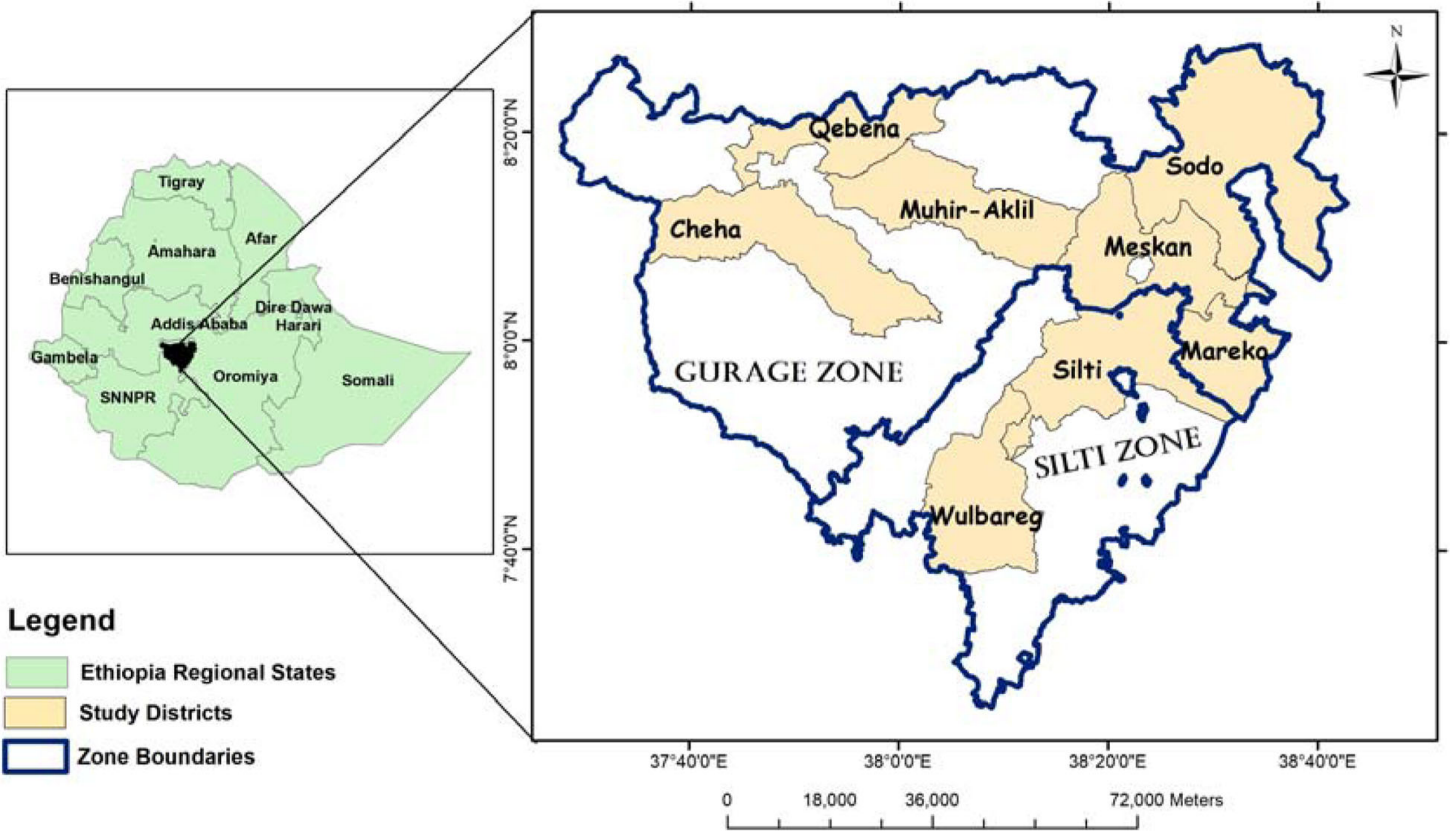
Map of Ethiopia and the study districts in the respective zones

Gurage and Silti zones are situated in Southern Nations, Nationalities and Peoples Region (SNNPR) and located in south central Ethiopia. Gurage zone is located at 7° 40′ 0″–8° 30′ 0″ N and 37° 50′ 0″–38° 40′ 0″ E with altitudinal range stretching between 1000 and 3600 m a.s.l. and covers an area of 5893.5 km^2^ [19]. On the other hand, Silti zone is located at 7° 40′ 0″–8° 10′ 0″ N and 37° 50′ 0″–38° 40′ 0″ E with altitudinal range stretching between 1640 and 3277 m a.s.l. and cover an area of 2537.5 km^2^**.** The geographical locations of the two zones and the study districts are shown in Fig. 1.

**Fig. 2:**
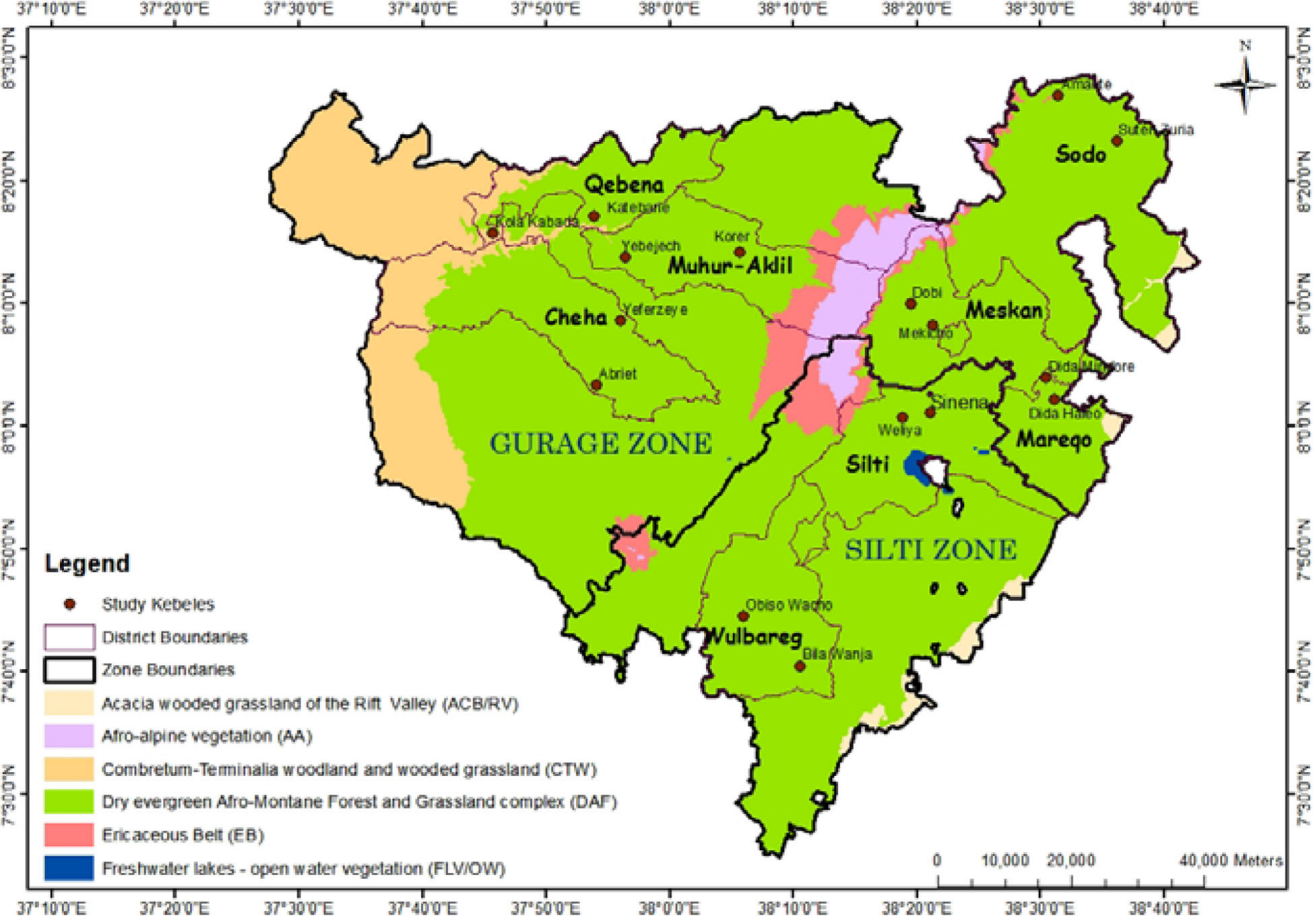
The vegetation types of Gurage and Silti zones (based on map in Atlas of the Potential Vegetation of Ethiopia by Friis et al. (2011))

Gurage zone consists of people belonging to the Gurage (Cheha, Meskan, Muhir-Aklil, and Sodo districts), Qebena, and Mareqo ethnic groups. These ethnic groups speak Guragigna, Qebena, and Libido languages, respectively. Silti zone is comprised of people identified as the Silti ethnic group, and Siltigna is the spoken language. In the present study, this ethnic group is represented by Silti and Wulbareg districts. The geographic proximity between Gurage and Silti zones led to extensive intermarriage between the four ethnic groups (Gurage, Silti, Mareqo, Qebena) and maintain inter-ethnic contacts [20]. This mutual influence has been shown in the respective ways of living which most likely resulted from this cultural mix [20]. Extensive cultivation of Enset (*Ensete ventricosum* (Welw.) Cheesman), traditional housing, artifacts, and mode of production are the best-known shared cultural practices of the zones and at large in SNNPR [21]. Enset is the main food crop together with *Hordeum vulgare L. Waif*. (barley), pulses, potatoes, and cabbage. The major cash crops are *Catha edulis* Forsk (Khat), *Coffea arabica* (Buna), *Eragrostis tef* (Zucc.) Trotter (Teff), and *Guizotia abyssinica* (L. f.) Cass (Noug). Animal husbandry is also part of subsistence farmer’s way of life. Amharic language is widely spoken by the communities, and sometimes, it is used as the lingua franca in the zones. Based on the recent classification of potential vegetation types as described in Friis et al. [22], the study area is dominantly characterized by the dry evergreen Afromontane forest and grassland complex (the undifferentiated Afromontane forest subtype) (Fig. 2).

### Informant selection and data collection

Ethnobotanical surveys were conducted between May and October 2016. A total of 320 informants (40 from each study district) were selected randomly following Gomez-Beloz, [23]. Semi-structured interviews during walk-in-the-woods or by using plant “props” (freshly collected plant material or photographs), focus group discussions, participant observation, and market survey were used for collecting data following the methods described [24–26]. Ethnobotanical information including list of all medicinal plants and parts used, kind of humans/livestock ailments treated, and medicinal plants that are sold in the local market were documented. Interview was conducted in Amharic and local languages. Written permission to conduct the research was obtained from the respective zone and district administrative officials. Prior informed consent was obtained from each informant before every interview.

### Plant collection and Identification

All plant specimens were collected, dried, identified, and deposited in the National Herbarium (ETH) of Addis Ababa University. Identification was made by using Flora of Ethiopia and Eritrea [27–34], in comparison with authenticated specimens from the herbarium and later confirmed by senior taxonomists of the herbarium.

### Data analysis

Corrected fidelity level (rank order priority) was used to identify the most culturally important medicinal plants from each study districts [35]. Rank order priority (ROP) was calculated using the formula: ROP = FL × RPL. Fidelity level (FL) is used to quantify the percentage of informants confirming the use of a plant species for the same major purpose. It is computed as:

$$ \mathrm{FL}\left(\%\right)= Ip/ Iu\times 100 $$

where *Ip* refers to the number of informants who indicated a specific medicinal plant species is used to treat the same major ailment and *Iu* is the total number of informants who mentioned the plant for treating any ailment. A high FL value (near 100%) for a plant indicates that all of the use reports mentioned the plant for a specific treatment, regardless of the number of times mentioned, whereas a low FL value is obtained for plants that are used for many different purposes, and/or known by few informants. However, plants known by few informants for the treatment of limited number of ailments might have high FL values [36]. In addition, plants with similar FL values but known to different numbers of informants may vary in their healing potential. Therefore, to minimize such kind of inconvenience, only plants mentioned by at least three informants were considered. In order to differentiate the healing potential of plants with similar FL value a correlation index called Relative Popularity Level (RPL) was calculated [35, 37]. RPL has values ranged between 0 and 1. The range categorize the plants into “popular” (RPL = 1) and “unpopular” (RPL < 1) groups. Popular plants are cited by more than half of the total number of informants (for example in the case of Qebena where the highest number of informants that cite a plant for of any therapeutic effect is 30 informants. So, plants cited by 15 or more informants are considered as popular, RPL score equals to 1. RPL is given less than 1 for “unpopular” plants which are cited by less than 15 informants). Exact RPL values for “unpopular” plants were determined by dividing the total number of informants who mentioned the plant for treating any ailment to half of the maximum number of informants (Iu/15 in case of Qebena). Finally, ROP was calculated by multiplying FL values by RPL values.

The Jaccard similarity coefficient was used to compare medicinal plants and report similarity between the districts. In order to analyze the variability in medicinal plant species among the districts, two presence or absence matrices were created. The first matrix considered all medicinal plant species cited by informants, and the second matrix considered plant species that scored minimum rank order priority of 50% (ROP ≥ 50%). The similarity between any pair of study site in terms of medicinal plant species mentioned was calculated using the Jaccard similarity coefficient:

$$ JI=\frac{a}{a+b+c} $$

where JI is the Jaccard similarity index, “a” is the number of species shared by or common to any compared pair of study sites and “b” and “c” are the number of medicinal plant species reported solely in one study district (b for one study site and c for the other). JI values range between 0 and 1, whereby a value of 1 indicates complete similarity. Then, the similarity coefficient for each pair of study site was used to obtain a dendrogram using unweighted pair-group method analysis (UPGMA; links a new item to the arithmetic average of a group) [38]. Cluster analysis is generally used to group study sites into categories based on their dissimilarities or partition heterogeneous elements into relatively homogenous groups [39].

Informant consensus factor (ICF) was used to analyze intercultural variations of plant uses among different groups [9]. The factor obtained shows the consistency of informant’s knowledge about a particular remedy for a particular ailment. It was calculated following Heinrich et al. [40]:

$$ \mathrm{ICF}=\frac{nur- nt}{nur-1} $$

where *nur* refers to the number of use reports of an informant for a particular ailment category and *nt* to the number of species used for a particular illness.

## Results

### Medicinal plants used to treat human ailments

A total of 213 plant species belonging to 79 families and 175 genera, and a fungus species were documented and collected (Additional file 1). In terms of percentage of plant species, the family Asteraceae (25 spp., 12%) appeared to be the most dominant plant family followed by Lamiaceae (20 spp., 10%), Fabaceae (11 spp., 5%), Euphorbiaceae (9 spp., 4%), and Solanaceae (9 spp., 4%).

### Ethno veterinary medicinal plants diversity

The medicinal plants used to treat livestock disease consisted of 95 species, in 82 genera and 48 families (Additional file 2). The most commonly mentioned plant families containing ethnoveterinary species were Asteraceae (11 spp., 12%), Lamiaceae (8 spp., 9%), Solanaceae (8 spp., 9%), and Fabaceae (6 spp., 7%). Among these species, 26% (64 spp.) were used against diseases of both human and livestock and 13% (31 spp.) were employed to treat diseases of livestock only.

### Relative healing potential of medicinal plants

Relative healing potential of medicinal plants was computed for all reported medicinal plants. Eighty species were identified as the most preferred plants (ROP ≥ 50%). The list of most important species (ROP ≥ 50%) along with their use categories and reported study district are provided in Additional file 3. *Ajuga integrifolia*, *Clerodendrum myricoides*, *Hagenia abyssinica*, *Ruta chalepensis*, and *Solanum incanum* (ROP = 100%) had high fidelity level for treating infectious and intestinal parasitic diseases. Diseases of the digestive system were primarily treated by *Acacia seyal*, *Bridelia micrantha*, *Ficus vasta*, *Maytenus heterophylla*, *Myrica salicifolia*, and *Verbena officinalis* (ROP = 100%). Respiratory system diseases were mainly cured by *Catha edulis*, *Ocimum lamiifolium*, and *Pittosporum viridiflorum* (ROP = 100%). The genitourinary ailments were cured using *Foeniculum vulgare* (ROP = 95%) and *Lepidium sativum* (ROP = 88%); diseases of the musculoskeletal system *by Ajuga integrifolia* (ROP = 100%); diseases of the skin and subcutaneous tissue by *Argemone mexicana*, *Plantago lanceolata*, and *Salvia nilotica* (ROP = 100%); rabies mostly treated by *Phytolacca dodecandra* (ROP = 73%); *Carica papaya* (ROP = 100%) used to cure malaria. For the category of dental and oral problems, *Ekebergia capensis and Datura stramonium* (ROP = 100%) were found to be the most important. Liver complaints were mainly treated by *Ensete ventricosum* (ROP = 84%), *Justicia schimperiana* (ROP = 100%), and a fungus sp. *Calvatia* sp. (Agaricaceae) (ROP = 100%); inflammations related to anthrax were mostly treated by *Brassica nigra* (ROP = 100%) and *Polygala sadebeckiana* (ROP = 84%).

### Similarity among the community based on all cited medicinal plants

Computed dissimilarity coefficient using all cited medicinal plants was above 0.5 (Table 1). In relative term, Mareqo and Muhir-Aklil showed the smallest similarity (JI = 0.26), and the highest similarity (JI = 0.47) was obtained between Silti and Wulbareg districts.

**Table 1:** Similarity of medicinal plant species cited among the study districts

	Sodo	Muhir-Aklil	Cheha	Silti	Wulbareg	Qebena	Mareqo	Meskan
Sodo	1							
Muhir-Aklil	0.40	1						
Cheha	0.39	0.42	1					
Silti	0.37	0.38	0.34	1				
Wulbareg	0.35	0.27	0.35	*0.47*	1			
Qebena	0.31	0.34	0.38	0.34	0.37	1		
Mareqo	0.35	*0.26*	0.30	0.45	0.41	0.35	1	
Meskan	0.37	0.35	0.37	0.42	0.34	0.32	0.34	1

Jaccard similarity indices (0–1), 1 = similar; 0 = dissimilar. Note that the matrix is symmetrical about the diagonal

**Fig. 3:**
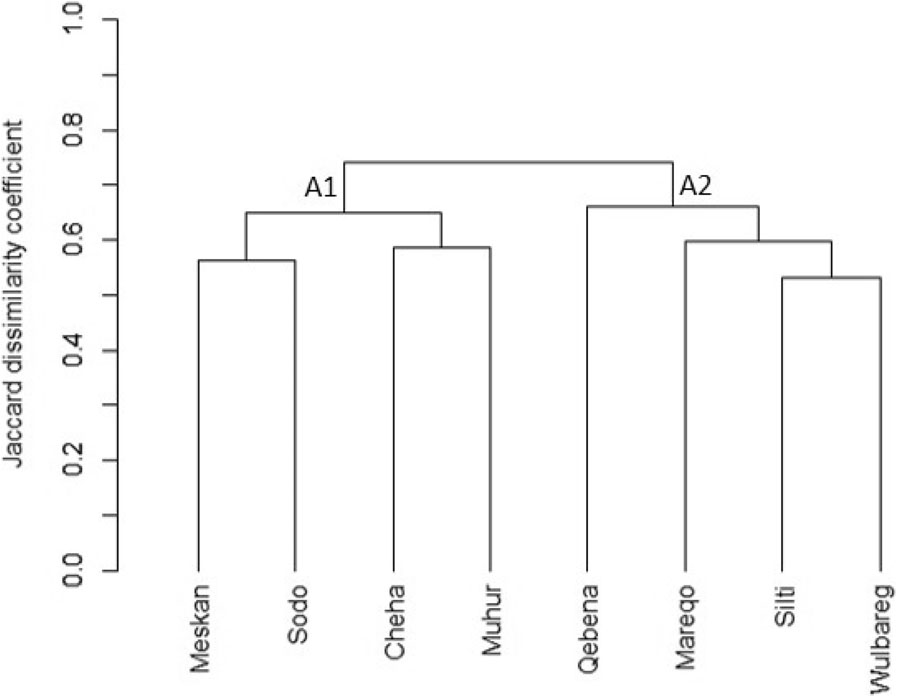
Dendrogram showing the dissimilarity between study districts based on all medicinal plant species mentioned (based on Jaccard dissimilarity coefficient and UPGMA clustering method)

High cophenetic correlation coefficient (0.92) was obtained using the UPGMA (average) clustering method, unlike the single, complete, and ward methods that had cophenetic correlation coefficient of 0.90, 0.88, and 0.73, respectively. High cophenetic correlation coefficient indicates that the resulting dendrogram is a good fit of the reality. The dendrogram formed, using UPGMA (average) clustering method, clustered the study sites broadly into two groups that are fairly close (Fig. 3). In the first cluster A1, grouped Meskan, Sodo, Cheha, and Muhir-Aklil as deemed most similar. In cluster A2, Qebena, Mareqo, Silti, and Wulbareg were grouped as similar or closely related. The first two sites in cluster A2 (Qebena and Mareqo) were outside the sub-cluster formed between Silti and Wulbareg.

### Similarity among the community based on culturally most important medicinal plants (ROP ≥ 50%)

Similarity coefficient between study communities based on culturally most important medicinal plants range between 0.10 and 0.45 (Table 2). In relative term, the most dissimilar study sites were Muhir-Aklil and Wulbareg (JI = 0.10). The highest similarity was obtained between Cheha and Qebena districts (JI = 0.45).

**Table 2:** Similarity of culturally most important medicinal plant species among the study

	Sodo	Muhir-Aklil	Cheha	Silti	Wulbareg	Qebena	Mareqo	Meskan
Sodo	1							
Muhir-Aklil	0.12	1						
Cheha	0.11	0.30	1					
Silti	0.12	0.19	0.22	1				
Wulbareg	0.15	0.10	0.20	0.33	1			
Qebena	0.13	0.29	0.45	0.23	0.18	1		
Mareqo	0.14	0.19	0.25	0.43	0.24	0.27	1	
Meskan	0.31	0.27	0.30	0.34	0.14	0.20	0.29	1

Jaccard similarity indices (0–1), 1 = similar; 0 = dissimilar. Note that the matrix is symmetrical about the diagonal

**Fig. 4:**
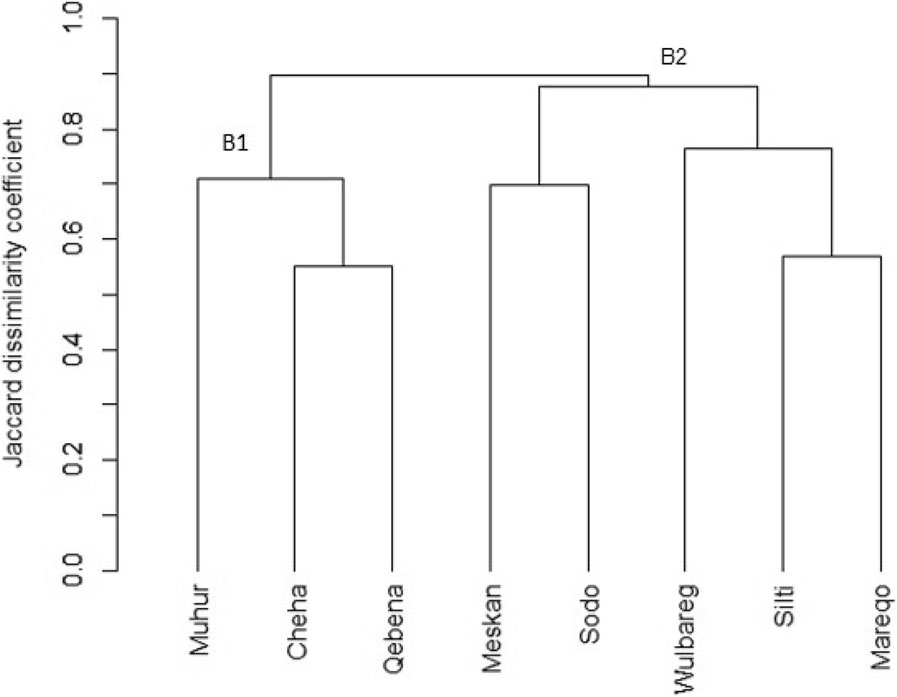
Dendrogram showing the dissimilarity between study sites based on important medicinal plant species (based on the Jaccard dissimilarity coefficient and UPGMA clustering method)

Cophenetic correlation coefficient obtained using UPGMA, Complete, Ward, and Single methods were 0.78, 0.77, 0.74, and 0.71, respectively. Based on important medicinal plant species, the dendrogram obtained from the UPGMA (average) clustering method also grouped the study districts into two clusters but the grouping differ considerably from the one obtained using all cited plant species (Fig. 4). In cluster B1, Cheha and Qebena are grouped into the same sub-cluster while Muhir-Aklil stands alone on the same branch. On the other hand, in cluster B2, Meskan and Sodo were clustered together, also Silti and Mareqo on the same branch with Wulbareg as an out-group.

### Informant consensus factor (ICF)

The ailments reported were grouped into 19 main categories (Additional file 4). Overall, a high level of consensus among informants (mean ICF value = 0.73) regarding ailments treated by medicinal plants was found (Table 3). The high value was obtained for infectious and intestinal parasitic diseases, diseases of the respiratory system, and other unclassified in all study districts (ICF = 0.67–0.91). Diseases of the digestive system (ICF = 0.69–0.86 in most districts) and liver complaints (ICF = 0.64–0.92 in most districts) were culturally accepted being treated effectively with medicinal plants and have also scored relatively higher ICF values.

**Table 3:** Informant consensus factor (ICF) values of each study districts

Major use categories	Study districts								Average
	C	MA	ME	SO	Q	MQ	SI	W	
Diseases of the musculoskeletal system (DMS)	*0.86*	*0.76*	0.91	0.82	0.89	0.9	0.84	0.85	0.85
Diseases of the ear and mastoid process (earache) (DEM)	–	–	–	–	–	–	0.93	1	0.97
Other unclassified (OUH)	*0.88*	*0.86*	*0.81*	*0.73*	*0.9*	*0.85*	*0.81*	*0.79*	0.83
Liver complaints (LC)	0.64	*0.75*	*0.77*	*0.79*	0.92	0.92	0.92	0.89	0.83
Inflammation related to the anthrax (IRA)	0.82	*0.78*	–	–	*0.93*	0.75	–	–	0.82
Diseases of the respiratory system (DRS)	*0.85*	*0.74*	*0.76*	*0.67*	*0.91*	*0.83*	*0.81*	*0.7*	0.78
Infectious and intestinal parasitic diseases (IIP)	*0.71*	*0.73*	*0.79*	*0.72*	*0.9*	*0.86*	*0.82*	*0.74*	0.78
Pregnancy, childbirth, and the puerperium (PCP)	0.73	–	0.71	0.64	–	0.82	0.78	0.76	0.74
Diseases of the digestive system (DDS)	*0.69*	*0.74*	*0.86*	0.53	*0.85*	*0.78*	*0.78*	*0.63*	0.73
Diseases of the genitourinary system (DGS)	0.83	0.38	0.56	0.33	*0.92*	1	0.85	0.83	0.71
Dental and oral diseases (DOD)	*0.8*	0.63	0.62	0.5	0.83	0.8	*0.61*	*0.75*	0.69
Headache, fever, and malaria (HFM)	0.68	0.71	0.63	0.43	*0.92*	*0.76*	0.7	*0.68*	0.69
Diseases of the skin and subcutaneous tissue (DSS)	*0.73*	0.59	*0.56*	*0.68*	*0.75*	0.66	*0.78*	*0.68*	0.68
Diseases of the eye and adnexa (DEA)	0.38	0.64	0.7	0.71	0.5	–	0.62	0.5	0.58
Injury, poisoning, and certain other consequences of external causes (IPE)	–	–	0.54	0.68	–	–	0.44	–	0.55
Diseases of the circulatory system (DCS)	0.38	0.4	–	0.4	–	1	0.3	–	0.50
Livestock									
Ectoparasites (livestock ailments) (ECL)	0.71	0.81	1	0.7	0.91	1	0.83	0.58	0.82
Livestock (infectious and parasitic diseases) (LIPD)	0.61	*0.6*	*0.77*	0.64	0.75	0.89	*0.78*	0.46	0.69
Others (livestock) (OL)	0.7	0.54	*0.71*	*0.7*	0.56	0.81	0.77	0.5	0.66
								Average	*0.73*

Study districts: Cheha (C), Muhir-Aklil (MA), Meskan (ME), Sodo (SO), Qebena (Q), Mareqo (MQ), Silti (SI), and Wulbareg (W); ICF value ranges between 0 and 1, value close to 1 indicates high level of informant consensus; italics with number of use reports greater than half of use report in the study group and considered here as high ICF score in the district, “–” the ailment was not mentioned

### Marketability of medicinal plants

Few medicinal plants were being sold in six open markets visited. These medicinal plants are commonly used and well known by the local people. The medicinal plants encountered in the market places were sold or bought for medicinal and non-medicinal uses. Across the study districts, six herbs (*Artemisia afra*, *Hagenia abyssinica*, *Lepidium sativum*, *Polygala sadebeckiana*, *Satureja abyssinica*, and *Silene macrosolen*) were solely sold for their medicinal values and used as a source of income (Table 4). All of the medicinal sellers encountered were women. The women pointed out that they either collect the plant parts from their own garden, purchase from medicinal plant sellers, or collect from nearby forest patches in the zones. For example, *Silene macrosolen* which is reported to grow mainly in the highlands of Meskan District is sold to the sellers in Agana (near to Cheha), Butajira (Meskan), and Bui (Sodo) open markets. *Satureja abyssinica* also grows in highland areas of Cheha and Meskan districts, and the people of Silti district procure the plant from Qebet open market found in the area. With regard to medicinal plants trading, a handful or a cup of medicinal plant parts (leaves or seeds) cost a minimum price exchange of 5 birr (0.14$). An informant in Mareqo district reported that a practice of cultivating and trading half a kilo of *Jatropha curcas* seeds to a neighboring healer living in Silti District worth 20 birr (0.6$).

**Table 4:** Checklist of plant species used only for their medicinal values and sold in the open markets visited in the study area

Species	Open markets						
	Agena	Bui	Butajira	Hawariyat	Imdiber	Qibet	Qoshe
*Artemisia afra* Jacq. ex Willd. [Asteraceae]*	√		√				√
*Hagenia abyssinica* (Bruce) J.F. Gmel. [Rosaceae]	√		√		√		
Lepidium sativum L. [Brassicaceae]*	√	√	√	√	√	√	√
*Polygala sadebeckiana* Gurke [Polygalaceae]	√			√	√		
Silene macrosolen A. Rich. [Caryophyllaceae]	√	√	√		√		
*Satureja abyssinica* (Benth.) Briq. [Lamiaceae]	√		√				

Agena market—near to Cheha and Qebena; Bui market—Sodo; Butajira market—Meskan; Hawariyat market—Muhir-Aklil; Imdiber market—Cheha; Qibet market—Silti; Qoshe market—Mareqo

### Medicinal plants naming—ethnotaxonomy

Nomenclature of medicinal plant sometimes involves meaning related to the plant use or other suggestive information of the plants. This was revealed in the local names of 23 medicinal plant species; 4 of the local names reflect medicinal uses, and the remaining 19 species indicate morphological character (growth form, truck color, and leaf shape), as being poisonous, and taste and smell of the plants (Table 5). Seventy-nine percent of the medicinal plant species have local names in one or more local languages of the studied districts that are also sometimes used similarly or with a little differed intonation among the communities. In a few cases, one local name is used for many species that have similar medicinal use. For example, local name *Kureshe* was used for *Crinum abyssinicum*, *Sauromatum venosum*, and *Tacca leontopetaloides* which are used to cure livestock ailments (anthrax/blackleg).

**Table 5:** Local names of medicinal plants and their meaning (S–Silti; G–Guragigna; M–Mareqo; Q–Qebena), “*”–endemic

Scientific name [Family]	Local name	Direct meaning of the local name in English	Meaning reflecting
*Acacia abyssinica* Hochst. ex Benth. [Fabaceae]	Teme-gerar (S, G)	Black acacia	Trunk color
*Acacia seyal* Del. [Fabaceae]	Wacho-gerar (S, G)	Red acacia	Trunk color
*Ajuga integrifolia* Buch-Ham. [Lamiaceae]	Anamuro, ema telit (G)	Makes an infant hate breast feeding, used purposefully to stop breast feeding	Bitter taste
*Aloe pubescens* Reyonolds. [Aloaceae]*	Merdedeye (G)	Saw like (a tool with toothed blade)	Marginal teeth of the leaf
*Artemisia abyssinica* Sch. Bip. ex A. Rich. [Asteraceae]	Chiyanchiye (G)	Bad smell	Leaves with bad smell
*Brucea antidysenterica* J. F .Mill. [Simaroubaceae]	Yemoyet bosha (G)	Leaves of *moyet* (a social group known to exist in Gurage) that is used during cultural ceremony	Ceremonial use
*Clematis simensis* Fresen. [Ranunculaceae]	Yegawa wedero (G)	Fool’s rope	Climber growth form
*Convolvulus sagittatus* Thunb. [Convolvulaceae]	Minen debo (M)	Medicinal	Medicinal use
*Crotalaria incana* L. [Fabaceae]	Meza qutel (G)	Leave for wound	Medicinal use
*Cucumis ficifolius* A. Rich. [Cucurbitaceae]	Hulgerecho (M), Adene debaqula (Q), Yemeder qimbiba, Yafer-granger (G), Yale-tay (S)	Monkey’s genital organ (Q); Fruits running on the ground (G),	Fruit shape and growth form/arrangement
*Cynoglossum coeruleum* Hochst. ex A.DC. [Boraginaceae]	Yitebtiye (G), Bertetusa (Q), Hatemaqo (Q, S)	Sticky	The sticky nature of the fruits
*Cyphostemma niveum* (Hochst. Ex Schweinf.) Desc. [Vitaceae]	Yeseb eje (G)	Human hand	Leaf shape
*Foeniculum vulgare* Miller [Apiaceae]	Wet-ambo (G)		Stems hollow when mature
*Fuerstia africana* T.C.E. Fr. [Lamiaceae]	Yegiye insosla (G), Nazoli (S), Hureda (M)	Insosla (*Impatiens tinctoria*) which is a dark red dye extracted from the tubers used as beauty treatment, *Fuerstia africana* is called for dog’s which is not similar to above mentioned species (G)	Color of the red juice squeezed from the leaves
*Haplocarpha schimperi* (Sch. Bip.) Beauv. [Asteraceae]	Ayene beda (G)	Takes the eyes	Poisonous to the eyes
*Pavonia urens* Cav. [Malvaceae]	Menatef (G)	Trigger vomiting when feeling sick	Medicinal use
*Polygala sadebeckiana* Gurke [Polygalaceae]	Shime-itere chiza(G), Shime yeter zebo (Q), Qiteriye(G)	Local name of antrax/blackleg (G, Q); Finger like (G)	Medicinal use, root structure
*Plantago lanceolata* L. [Plantaginaceae]	Yefur enzir (G)	Rat’s ear	Leaf shape
*Rhoicissus tridentata* (L. f.) Wild & Drummond [Vitaceae]	Yegawa wedero (G), dubi fizuta (Q)	Fool’s rope	Climbing nature
*Rhynchosia minima* (L.) DC. [Fabaceae]	Yefur enzir (G)	Rat’s era	Leaf shape
*Thunbergia ruspolii* Lindau [Acanthaceae]*	Yangacha qomet (G)	Cat’s *Lagenaria siceraria*	Resemble fruit and flower (corolla) shape of *Lagenaria siceraria*
*Verbascum sinaiticum* Benth. [Scrophulariaceae]	Yemar enzir (G), Huleten huta (M), Yumar amel ( S)	Donkey’s ear	Leaf shape
*Xanthium strumarium* L. [Asteraceae]	Yetey-soohe (G), Gereba uta (M)	Sheep’s spine	Sticky nature of the fruit, usually seen sticking on sheep’s fur

## Discussion

There is a good agreement among the informants regarding therapeutic uses of reported medicinal plant species. Especially, the three use categories (infectious and intestinal parasitic diseases, diseases of the respiratory system, and other unclassified) scored high ICF values in all study districts. Different studies conducted in Ethiopia also reported high ICF value for the same illness categories [41–44]. These groups of ailments are reported as common in the study area and elsewhere in the country. This could reflect the fact that some frequently occurring ailments are usually treated by medicinal plants. Ailments (liver complaints, diseases of the digestive system) that were culturally accepted being treated effectively by using medicinal plants have also scored relatively higher ICF values as reported in various studies [45, 46].

High ROP value indicates good healing potential of a plant and the tendency of informants relying on specific medicinal plant species for treating the respective diseases reported [35]. In the present study, the number of plants (80 plant species, 33%) that scored ROP value greater than 50% is considerably smaller than that of plants with ROP < 50% (164 plant species, 67%), despite the fact that these plants are with more frequent uses. This may be probably due to the diminishing popularity of many of the herbal medicine used among the study groups as stated in Friedman et al. [35]. It is also probable that the people are becoming selective and only use plants that are accepted in the culture as being more effective. Plants with computed ROP value at least 50% are referred here as culturally important medicinal plant species. Of 80 culturally important plant species, 37 species attained ROP values equals to 100% and might be taken as highly regarded and widely used in the community (see Additional file 3). These plants are widely known in the community and believed to be more effective [44, 46]. Baydoun et al [46] reported that medicinal plants with high FL score reveal the outstanding choice of informants for treating specific illness. High FL also indicates the similarity of use reports for a given species whereas low FL are obtained for plant species that are used for many different purposes [44]. In these use categories, high number of medicinal plants is used in the treatment of abdominal pain. This could indicate the high prevalence of the disease in the study districts and presence of shared knowledge among the communities to cure frequently occurring ailments. Indeed, this supports the mere fact that a problem leads to a solution.

Computed dissimilarity coefficient using all cited medicinal plants was above 0.5, reflecting the fact that co-presence of plant species cited was less than 50%. By using this value, the clustering displayed the similarities between the studies sites as mostly represented in the terms of ethnicity. This indicated that the same ethnic group generally shared many medicinal plants. Ethnic groups are defined as relating to large groups of people classed according to common racial, tribal, religious, linguistic, or cultural origin or background [47]. This historical linkage in the study communities is shown in the vestiges of mutual influence in medicinal plant application in one or the other way. This was seen in the first dendrogram (Fig. 3), in which districts from same ethnic groups were grouped together. Cheha, Muhir-Aklil, Meskan, and Sodo from Gurage ethnic groups; Silti and Wulbareg from Silti ethnic group; Qebena and Mareqo districts stands alone and representing Qebena and Mareqo ethnic groups, respectively. Several studies have emphasized that ethnic, social, cultural, and geographical factors are the main controllers of the number of species used by communities [13, 48, 49]. In other way, as indicated in previous studies, plant use knowledge can be passed down from generation to generation vertically after being taught by a family member (ancestral knowledge) or horizontally by sharing of information between peers [50–52]. One can assume from the present study that most of the free-listed plant knowledge have been passed down from their ancestors. In this case, the differences obtained in all listed plants known by the community in the respective districts could be resulted from the route of knowledge transfer or it has ethnic origin touch.

Dendrogram obtained based on culturally important plants is completely different from the one based on all cited plants. Unlike the first, it ignores ethnic reflection. Thus, the pattern of distribution of culturally important medicinal plant species (plants that scored ROP ≥ 50%) across the respective study sites could provide grounds to make further points for consideration and to infer about the way in which these plant species are being used in the community. In the cluster B1 (Fig. 4), Cheha and Qebena are grouped into the same sub-cluster while Muhir-Aklil stands alone on the same branch, whereas cluster B2 clustered Meskan and Sodo districts together, and also Silti and Mareqo districts on the same branch with Wulbareg as an out-group. Here it could be suggested that ethnic background has minor effect on the differences in clustering. Rather, factors such as close vicinity and geographical orientation of the respected study sites seem to have an important influence on culturally important medicinal plant species. The clustering result suggested that groups that are geographically close often exchange information on most important plants. This would mean that with time, communities living in an area irrespective of their ethnicity exchange knowledge that is vital for survival. From the second cluster, the plant knowledge usually shared are actively used, believed to be tested through time and considered effective in the community. The influence of proximity is further explained by informants in which communities in Wulbareg district have a long historical relationship with communities of Alaba district (a different ethnic group not included in the present study) and lived in close proximity. Consequently, the fact that Wulbareg stands as an out-group in the second cluster might be supposed that communities in Wulbareg exchange medicinal plant use knowledge with the people of Alaba district living in close proximity than their relatives in Silti. Geographical proximity of communities as the most influential factor of similarities in the use of medicinal plant species was also stated in the findings of Coe [53] and Avocèvou-Ayisso et al. [9]. In general, different studies ascertain that local availability of plant species, specific environmental condition, and indigenous knowledge of a particular ethnic group mainly determine the medicinal plant lore of a community [54, 55]. Through time, however, knowledge exchange that can occur through friendships, kinship, inter-ethnical marriages, and togetherness alter medicinal plant lore of a community [56]. This fact is recognized by the informants in which groups live in nearby areas have mutual attraction than with areas relatively far irrespective of their ethnic belongingness.

Local languages are essential for transferring locally specific traditional knowledge that is vital for conserving the local environment and respective uses [57]. Local plant names occasionally had information for understanding their use or other property of the plants. This was reflected in the present study on some species (4 plant species) that were named after the ailment which the plants are used against, and few more names (19 plant species) indicate the morphology (growth form, truck color, and leaf shape), as being poisonous, or taste and odor. The naming system using the disease treated and morphological characters was similarly reported in different ethnobotanical studies conducted in Ethiopia [6, 42].

## Conclusions

Existing patterns of medicinal plant use vary among the studied districts as a function of combined effect of socio-cultural acceptance (through exchange of information), geographic proximity between groups, and market access. As a result, in spite of medicinal plant availability in the locality, plants that are widely accepted in the culture have higher ROP values, are best preferred, and are sold in the local markets where most can have easy access to find. Culturally, most important plants are widely used and best shared with nearby communities, and it could imply current (new) knowledge being practiced in the community. Unlike plants which are less frequently used by the community, in which plant use knowledge is conserved in the society (same ethnic group) and mostly not shared. Therefore, this study recommends undertaking detailed studies across different cultures and identification of mostly shared and preferred medicinal plants for further phytochemical and pharmacological research.

## Supplementary information

**Additional file 1.** List of medicinal plants used to treat human ailments: scientific name; plant family; vernacular name; growth form; plant parts used; ailment treated; methods of preparation / and additives (if any); routes of administration; study sites; voucher number

**Additional file 2.** List of medicinal plants used to treat livestock ailments: scientific name, family; vernacular name; growth form; plant parts used; ailment treated; methods of preparation; routes of administration; study sites; voucher number

**Additional file 3.** Rank order priority (ROP) values of culturally important species (ROP ≥50%) major uses, relative popularity level and study sites

**Additional file 4.** Major use categories and list of ailments/symptoms include (for compurting ICF value)

## Data Availability

All data generated or analyzed during this study are included in this published article and its supplementary information files are attached as Additional files 1, 2, 3, and 4.
