# Supplementary material for: Medicinal plant use practice in four ethnic communities (Gurage, Mareqo, Qebena, and Silti), south central Ethiopia
Source: J Ethnobiol Ethnomed. 2020 May 24;16:27. doi: 10.1186/s13002-020-00377-1 (PMC7245860; doi:10.1186/s13002-020-00377-1)
Supplement: Supplementary file 1 — Additional file 1. List of medicinal plants used to treat human ailments: scientific name; plant family; vernacular name; growth form; plant parts used; ailment treated; methods of preparation / and additives (if any); routes of administration; study sites; voucher number [file 13002_2020_377_MOESM1_ESM.docx]

Additional file 1. List of medicinal plants used to treat human ailments: scientific name; plant family; vernacular name; growth form; plant parts used; ailment treated; methods of preparation / and additives (if any); routes of administration; study sites; voucher number.

**Key:** Vernacular name: Guragigna (G); Qebena (Q); Mareqo (M); Silti (S); Amharic (A). Growth form (GF) - Tree (T); Shrub (S); Herb (H); Climber (C), Epiphyte. Part used (PU) -Leaf, L; Root, R; Fruit, Fr; Exocarp, Ex; Bark, B; Stem, St; Root bark, Rb; Flower, Fl; Bulb, Bu; Seed, Se; Corm, C; Tuber, Tu; Rhizome, Rh; Latex, Lat. Methods of preparation (MOP): Extract the Juice- Squeezed with little water added; Crushed – plant part material pounded/powdered; Infusion- soaked in water and filter; Decoction-boiled in water and filtered. Routes of administration (ROA). Study sites-Cheha (C); Qebena (Q); Wulbareg (W); Mareqo (MQ); Meskan (ME); Muhir-Aklil (MA); Silti (SI); Sodo (SO). Bold - Endemic, “*”-Cultivated.

| **Scientific name [Family]** | **Vernacular name (Local language)** | **GF** | **PU** | **Ailment treated (Local name)** | **MOP and Mixed plants or additives (if any)** | **ROA** | **Study sites** | **Voucher No.** |
| --- | --- | --- | --- | --- | --- | --- | --- | --- |
| *Hypoestes forskaolii (*Vahl) R. Br. * [Acanthaceae] | Yete beder (G) | H | L | Anemia, Amoebiasis, Gonorrhea (Emate), Afterpains, Stabbing pain, Anthrax (Shem-itere), Retained placenta, Menstrual pain, General health (increase weight, improve strength and boost infants immunity) | Decoction   - Mixed with butter, sugar or cooked with porridge | Oral | C, Q, MQ, ME, SI, SO | AT100 |
| *Justicia schimperiana* (Hochst. ex Nees) T. Anders. [Acanthaceae] | Abegafuye, Hneba (G), Temuga (S, M) | S | L | Liver complaint (Qoya, Seme dinku), Malaria; General malaise (Michi), | Infusion/ Decoction  -With Yogurt | Oral | All sites | AT106 |
|  |  |  |  | Swelling, Rheumatic pain, Lose ability to move | Boiled | Steam bath |  |  |
| ***Thunbergia ruspolii*** Lindau [Acanthaceae] | Yangacha qomet, Afuakiyi (G) | H | L, R | Abdominal pain, Diarrheal disease (Child) (Ye-dengiya-qar or Ye-sequriye (Gu), cholera, Liver complaint (Qoya, Seme dinku), General malaise (Michi), Hemorrhoid, General health (increase weight, improve strength and boost immunity of infants) | Infusion/Decoction | Oral | C, MA, SO | AT200 |
| *Agave sisalana* Perrine ex Engl. [Agavaceae] | Alage (A) | S | L, R | Evil spirit (Dorer, Likift, Buda), General malaise (Michi) | Boiled | Steam bath | ME, SI, W, SO | AT8 |
|  |  |  | L | Abdominal bloating | Infusion | Oral |  |  |
| *Allium cepa* L. [Alliaceae] * | Besh-shinkurt (G) | H | Bu | Retained placenta | Crushed | Oral | Q, MA, SO | AT12 |
| *Allium sativum* L. [Alliaceae]* | Tuma (G) | H | Bu | General malaise (Mich), Malaria, Abdominal pain | Decoction/  Crushed | Oral | All sites | AT13 |
|  |  |  |  | Common cold, coughing, Pneumonia (Sinbabie) | Decoction | Oral |  |  |
|  |  |  |  | Toothache | Crushed | Hold with teeth |  |  |
| ***Aloe pubescens*** Reyonolds. [Aloaceae] | Merdedeye, Yefuga gedel (G), Werab kershi (S) | H | R, L, Lat, | General health (increase weight, improve strength and boost immunity of infants), Stabbing pain,  General malaise (Mich), Swelling | Decoction | Oral | All sites | AT14 |
|  |  |  | Lat | Wound | Extract the latex (warm/cold) | Topical |  |  |
| *Achyranthes aspera* L. [Amaranthaceae] | Sha-she-megene (G) | H | L  L  L  L | Toothache | Crushed | Hold with teeth | MQ, MA, SI, | AT04 |
|  |  |  |  | General health (increase weight, improve strength and boost immunity of infants) | Decoction | Oral |  |  |
|  |  |  |  | Remove dirt from eyes | Extract the juice/ squeezed | Drop into the eyes |  |  |
|  |  |  |  | Skin burn | Crushed | Topical |  |  |
| *Amaranthus lividus* L.  [*Amaranthaceae*] | Meryit (G) | H | L | Indigestion (Qiter) | Boiled | Oral (eat) | MA | AT15 |
| *Cyathula uncinulata* (Schrad.) Schinz [Amaranthaceae] | Awesan kanfua (G) | H | L | Swelling, General malaise (Mich) | Boiled | Steam bath | SO | AT56 |
|  |  |  |  | Eye infection (Wucher) | Extract the juice/ squeezed | Drops into the eyes |  |  |
| *Lannea schimperi* (A. Rich.) Engl. [Anacardiaceae] | Abariyet (G) | T | Se | Dandruff (with sores on the scalp) | Crushed  -With butter | Topical | C | AT113 |
| Rhus retinorrhoea A. Rich. [Anacardiaceae] |  | S | L | Liver complaint (Qoya, Seme dinku) | Infusion | Oral | W | AT166 |
| *Rhus vulgaris* Meikle [Anacardiaceae] |  | S | St | Toothache | Crushed | Hold with teeth | Q | AT167 |
| *Schinus molle* L. [Anacardiaceae] | Trumantre (G) | T | Fr | Tonsillitis | Infusion | Gargle to rinse the throat | MQ, ME | AT180 |
| *Foeniculum vulgare* Miller [Apiaceae]* | Wet-ambo (G), Enshelal (Q), Aneshway (S), Ansho (M) | H | L | Urinary retention, Abdominal pain | Infusion/Decoction | Oral | All sites | AT85 |
|  |  |  |  | Epistaxis | Extract the juice/ squeeze | Nose drops |  |  |
|  |  |  |  | Evil spirit (Dorer, Likift, Buda), General malaise(Mich) | Infusion, boiled | Oral, Steam bath |  |  |
|  |  |  | R, L | Gonorrhoea (Emate) | Infusion/Decoction | Oral |  |  |
| *Acokanthera schimperi* (A.DC.) Schweinf. [Apocynaceae] | Adere (G) | T | L | Indigestion (Qiter) | Infusion | Oral | C, MA | AT06 |
| *Carissa spinarum* L. [Apocynaceae] |  | S | R | General malaise | Infusion | Oral | C, Q, MA, SI, SO | AT35 |
|  |  |  | L | Malnutrition | Crushed | Tied/paste |  |  |
| *Dregea schimperi* (Decne.) Bullock [Apocynaceae] |  | C | L | Ashma, Azurit | Infusion/decoction | Oral | SO | AT62 |
| *Colocasia esculenta* (L.) Schott [Araceae]* | Godelyi (A) | H | Tu | Anthrax (Shem-itere), Wound, Swelling | Decoction | Topical | C, MA | AT48 |
| *Asparagus africanus* Lam. [Asparagaceae] | Yefur ded (G), Siriti (M) | C | L | Herpes zoster | Crushed | Topical and expose to sun | C, MA, MQ, ME, SI | AT21 |
|  |  |  | R | Retained placenta | Decoction | Oral |  |  |
| *Acmella caulirhiza* Del. [Asteraceae] | Anshet (G), Afetego (A) | H | Fl, L, R | Tonsillitis | Infusion | Gargle to rinse the throat | All sites | AT05 |
|  |  |  |  | Toothache | Crushed | Hold with teeth |  |  |
| *Artemisia abyssinica* Sch. Bip. ex A. Rich. [Asteraceae] | Chekugne (A, G) Chiyanchiye (G), Chancho (S) | H | L | Coughing, Pneumonia (Sinbabie), Abdominal pain, Diarrhoea | Infusion | Oral | ME, MA, SI, SO | AT18 |
|  |  |  |  | Evil spirit (Dorer, Likift, Buda) | Infusion, Crushed | Oral, Smell |  |  |
| *Artemisia afra* Jacq. ex Willd. [Asteraceae]* | Naterar (G) | H | L | Headache, General malaise | Extract the juice/ squeezed | Nose drops | C, Q, MQ, ME, MA, SO | AT19 |
|  |  |  |  | Indigestion (Qiter) | Infusion | Oral |  |  |
| *Carduus schimperi* Sch. Bip. [Asteraceae] | Yete-soohe (G) | H | R | Diarrheal disease (Child) (Ye-dengiya-qar or Ye-sequriye (Gu), Hyperemesis, General health (increase weight, improve strength and boost immunity of infants) | Infusion /Decoction | Oral | MA | AT33 |
| *Conyza abyssinica* Sch. Bip. ex A. Rich. [Asteraceae] | Yefur ded (G) | H | L | Wound | Crushed | Topical | C, MA | AT51 |
| *Dicrocephala integrifolia* (L. f.) Kuntze. [Asteraceae] | Muachera, Bekulubash (G) | H | L | Abdominal pain | Infusion | Oral | C, SO | AT64 |
|  |  |  |  | Wound | Crushed | Topical |  |  |
| *Echinops hispidus* Fresen. [Asteraceae] | Yimar-soohe, Dender (G), umar soohe (Q) | H | R | Abdominal pain, Diarrhea | Infusion   - With Coffee/ tea | Oral | SI, SO | AT69 |
|  |  |  |  | Wound, Snake bite | Crushed | Topical |  |  |
|  |  |  | Fl | Swelling | Crushed | Topical |  |  |
| *Echinops kebericho* Mesfin. [Asteraceae]* | Chosa (G), Kebericho (S, M, Q) | H | R | Abdominal pain, Indigestion (Qiter) | Crushed/Chewed, infusion | Oral | All sites | AT70 |
|  |  |  |  | General malaise (Michi) | Burn | Smell the smoke |  |  |
| *Guizotia abyssinica* (L. f.) Cass. [Asteraceae]* | Nug (A) | H | Se | Coughing, Common cold | Roast, grind and drink the decoction | Oral | C, MA | AT91 |
| *Guizotia schimperi* Sch. Bip. ex Walp. [Asteraceae] | Mocho (A) | H | L | Dandruff (with sores on the scalp), Wound | Crushed | Topical | W, MA, SI | AT92 |
|  |  |  |  | General malaise (Michi) | Infusion | Oral |  |  |
| *Haplocarpha schimperi* (Sch. Bip.) Beauv. [Asteraceae] | Ayene beda (G) | H | R | Pyoderma “Wegfiy, Kofa” | Crushed | Topical | SO | AT94 |
| *Helianthus annuus* L. [Asteraceae]* | Suf (A) | H | Se | Coughing, Common cold | Decoction | Oral | MA | AT95 |
| *Helichrysum stenopterum* DC. [Asteraceae] |  | H | L | Eye infection (Wucher) | Extract the juice/ squeeze | Drops into the eyes | C | AT96 |
| *Lactuca inermis* Forssk. [Asteraceae] |  | H | L, R | Pyoderma (Wegfiy, Kofa), Common wart (Qintebiye, Foshe foshat) | Crushed | Topical | All sites | AT110 |
| *Laggera crispata (Vahl) Hepper & Wood. [Asteraceae]* | Ge-fon-foo (S) | H | L | Eye infection, To keep infected eye clean, Wound | Crushed | Topical, leaf also used to clean the eyes | C, ME | AT112 |
| *Microglossa pyrifolia (Lam.) O. Kuntze [Asteraceae]* | Chinar (G) | S | L | Toothache | Crushed/Chewed | Hold with teeth | C | AT129 |
| *Solanecio gigas* (Vatke) C. Jeffrey [Asteraceae] | Tonbi (G), Yezogare gaje (G, Q) | S | L, R | Liver complaint (Qoya, Seme dinku, Awezager), Retained placenta, Abdominal pain | Infusion | Oral | Q, MA | AT189 |
|  |  |  | L | Malaria | Infusion | Oral |  |  |
|  |  |  |  | Swelling | Crushed | Bath |  |  |
| *Solanecio mannii* (Hook. f.) C. Jeffrey [Asteraceae] | Gemar (G) | S | L | Liver complaint (Qoya, Seme dinku), Evil spirit (Dorer, Likift, Buda), | Infusion/ decoction | Oral | C, MA, SO | AT190 |
| *Tagetes minuta* L. [Asteraceae] | Chiyanchiye (G) | H | L | Wound | Crushed | Topical | Q, MQ, MA, | AT197 |
| *Vernonia myriantha* Hook. f. [Asteraceae] | Dengrita, Aguaje(G, Q) | S | L | Wound | Crushed | Topical | C, MA, ME, SO | AT205 |
| *Vernonia amygdalina* Del. [Asteraceae] | Gola (G), Heba (S, M) | S | L, St | Intestinal parasites, Abdominal pain, Malaria, Gastritis, Retained placenta | Infusion | Oral | All sites | AT206 |
| *Vernonia subligera* O. Hoffm. [Asteraceae] | Ereja (G) | S | L | Wound, Blood clotting | Crushed | Topical | Q, W, MA, SI | AT207 |
|  |  |  |  | Eye infection | Extract the juice/ squeeze | Drops into the eyes |  |  |
| *Vernonia theophrastifolia* Scheinf. Ex Oliv. & Hiern [Asteraceae] |  | S | L | Spider bite | Crushed | Topical | Q | AT208 |
| *Vernonia thomsoniana* Oliv. & Hiern ex Oliv. [Asteraceae] | Agunba (Q) | S | L | Malaria, Indigestion (Qiter) | Infusion | Oral | Q | AT209 |
| *Xanthium strumarium* L. [Asteraceae] | Yetey- soohe (G), Gereba uta( M) | H | L | Tinea versicolor (bechero), Pyoderma “Wegfiy, Kofa” | Crushed | Topical | MQ, ME | AT211 |
| *Balanites aegyptiaca* (L.) Del. [Balanitaceae] | Bedeno (A, G) | T | Lat | Headache | Infusion, Smoking | Nose drops, smell | MQ | AT22 |
| *Impatiens tinctoria* A. Rich. [Balsaminaceae] | Inshoshela (A) | H | L | Eye infection (Wucher) | Extract the juice/ squeeze | Drops into the eyes | Q, MA | AT101 |
|  |  |  |  | Indigestion (Qiter) | Infusion | Oral |  |  |
| *Stereospermum kunthianum* Cham. [Bignoniaceae] | Emequashiyet, Brete feje (G) | T | B | Indigestion (Qiter) | Infusion /Decoction | Oral | C | AT196 |
| *Cynoglossum coeruleum* Hochst. ex A.DC. [Boraginaceae] | Yitebtiye (G), Bertetusa (Q), Hatemaqo (Q, S) | H | L | General Malaise (Michi) | Extract the juice/ squeezed | Nose drops | C, Q, W, ME, MA, SI | AT58 |
|  |  |  |  | Wound | Extract the juice/squeezed | Topical |  |  |
| *Brassica carinata* A. Br. [Brassicaceae]* |  | H | Se | Anthrax (Shem- itere), Gastritis, Liver complaint (Qoya, Seme dinku) | Roast, grind and drink the infusion  - With egg yolk, milk or water | Oral | MA, SI | AT25 |
| *Brassica nigra* (L.) Koch [Brassicaceae]* | Senafich (A) | H | Se | Indigestion (Qiter), Amoebiasis | Grind and drink the infusion/use it as spice | Oral | C, Q, W, MQ, ME, SI, | AT26 |
| *Lepidium sativum* L. [Brassicaceae]* | Feto (A) | H | Se | Abdominal pain, Diarrhea, Amoebiasis, Gonorrhoea (Emate) Stabbing pain, Indigestion (Qiter), Headache, Evil spirit (Dorer, Likift, Buda), General malaise (Michi) | Infusion  -With Coffee/tea | Oral | All sites | AT117 |
|  |  |  |  | Toothache | Crushed | Hold with teeth |  |  |
|  |  |  |  | Dandruff (Fore fore) | Crushed | Topical |  |  |
| *Carica papaya* L. [Caricaceae]* | Papaya(A) | T | L, R | Malaria | Infusion/Decoction | Oral | C, Q, MQ, ME, MA, SI, SO | AT34 |
| *Silene macrosolen* A. Rich. [Caryophyllaceae] | Wegert (A) | H | R | Evil spirit (Dorer, Likift, Buda), repel snake | Burn | Smell the smoke | ME |  |
| *Catha edulis* (Vahl) Forssk. ex Endl. [Celastraceae]* | Chat (A) | T | L | Tonsillitis | Infusion | Oral | C, Q, MA, SI | AT36 |
| *Maytenus arbutifolia* (A. Rich) Wilczek [Celastraceae] | Cheryi (G), Konbol (S) | S | L | Tonsilitis | Infusion | Gargle to rinse the throat | W | AT125 |
| *Maytenus heterophylla* (Eckl. & Zeyh.) Robson [Celastraceae] | Cheryi (G) | S | L | Tonsilitis | Infusion | Gargle to rinse the throat | C, Q | AT126 |
|  |  |  |  | Epilipsy (Azurit) | Extract the juice/ squeezed | Oral |  |  |
| *Maytenus senegalensis* (Lam.) Exell [Celastraceae] | Cheryi (G) | S | L | Anthrax (Shem-itere) | Infusion/ Extract the juice | Oral/ Nose drops | C, Q, M | AT127 |
| *Chenopodium* sp. [Chenopodiaceae] | Amedmado (A) | H | L | Haemorrhoid | Crushed | Topical | C, SI | AT38 |
|  |  |  |  | Indigestion | Infusion | Oral |  |  |
| *Convolvulus sagittatus* Thunb. [Convolvulaceae] | Minen debo (M) | H | R | Diarrheal disease (Child) (Ye-dengiya-qar or Ye-sequriye (G), , Indigestion (Qiter) | Infusion | Oral | MQ | AT49 |
| *Convolvulus* cf. *kilimandschari* Engl. [Convolvulaceae] | Abeta (G) | C | R | Indigestion (Qiter), Abdominal pain | Infusion/decoction | Oral | ME, MA, SO | AT50 |
| *Ipomoea purpurea* (L.) Roth. [Convolvulaceae] | Abeta (G) | H | L, St | Diarrheal disease (Child) (Ye-dengiya-qar or Ye-sequriye (Gu), Abdominal pain, Sleeping problem (child) | Infusion | Oral | ME, SI | AT102 |
| *Kalanchoe densiflora* Rolfe [Crassulaceae] | Andahula (A) | H | L, R | Tonsilitis | Infusion | Gargle to rinse the throat | ME, MA, SO | AT107 |
|  |  |  | R | Abdominal pain (Children) | Infusion | Oral |  |  |
|  |  |  | L | Wound, To keep infected eye clean | Crushed (cold/warm) | Topical |  |  |
| *Kalanchoe* sp. [Crassulaceae] | Hanchuli (S) | H | L | General malaise(Michi), Swelling, Bone fracture, Eye infection | Crushed (cold/warm) | Topical | W | AT108 |
| *Cucumis ficifolius* A. Rich. [Cucurbitaceae] | Hulgerecho (M), Adene debaqula (Q), Yemeder qimbiba, Yafer-granger (G), Yale-tay (Si) | H | R | Anthrax (Shem-itere), Liver complaint (Qoya, Seme dinku), Abdominal pain, Diarrhoea, Indigestion (Qiter), Retained placenta | Infusion/  Decoction | Oral | All sites | AT54 |
|  |  |  | Fr | Lose ability to move, Tonsillitis | Infusion | Oral |  |  |
|  |  |  | R | Toothache | Crushed | Hold with teeth |  |  |
|  |  |  | R | Evil spirit (Dorer, Likift, Buda) | Infusion | Oral |  |  |
| *Cucurbita pepo* L. [Cucurbitaceae]* |  | H | Se | Mental disorder, Headache, Teniasis (Seto (K), Chima (G)) | Roast, grind and drink the infusion | Oral | C, Q | AT55 |
| *Kedrostis foetidissima* (Jacq.) Cogn. [Cucurbitaceae] | Neche tere (G) | H | L, R | Tonsilitis | Infusion | Gargle to rinse the throat | SI | AT109 |
|  |  |  | L, R | Herpes zoster | Crushed | Topical |  |  |
| *Lagenaria siceraria* (Molina) Standl. [Cucurbitaceae]* | Qomet (G) | H | L | Dandruff (with sores on the scalp), Wound | Crushed | Topical | C, Q, W, MQ, SO | AT111 |
|  |  |  | L | Rabies | Infusion | Oral |  |  |
| *Momordica foetida* Schumach. [Cucurbitaceae] | Araret, Tere (G, S), Yehonzet beye (G) | H | L | Wound, Dandruff, Evil spirit (Dorer, Likift, Buda) | Crushed | Topical | ME, MA, SI, SO | AT130 |
|  |  |  | L | Herpes zoster | Crushed | Topical |  |  |
| *Juniperus procera* Hochst. ex. Endl. [Cupressaceae] |  | T | L | Afterpains, Retained placenta | Decoction  -With milk | Oral | C, MQ, ME, SI, SO | AT105 |
| *Dioscorea alata* L. [Dioscoreaceae]* | Boiyna (A, G) | H | L | Tinea versicolor (Bechero) | Crushed | Topical | C, Q, MA | AT66 |
| *Euclea divinorum* Hiern. [Ebenaceae] | Migiyar, Mesa (G) | T | L | Indigestion (Qiter) | Infusion | Oral | C, SO | AT77 |
|  |  |  |  | Teniasis (Chima (G) Seto (K)) | Decoction | Oral |  |  |
|  |  |  |  | After pains (Stomach cramp) | Infusion | Oral |  |  |
| *Agarista salicifolia* (Comm. ex Lam.) Don [Ericaceae] | Adia (G) | S | B | Abdominal pain | Decoction | Oral | MA | AT09 |
| *Erica arborea* L. [Ericaceae] |  | S | L | Indigestion (Qiter), Abdominal bloating | Infusion | Oral | C, ME | AT75 |
| *Bridelia micrantha* (Hochst.) Baill. [Euphorbiaceae] | Anenebu, Qibeber (G) | T | B | Indigestion (Qiter) | Decoction | Oral | Q, W | AT27 |
| *Clutia abyssinica* Kaub. & Spach. [Euphorbiaceae] | Yemar semat (G) | S | L | TB (Neqeresa), Abdominal pain, Anthrax (Shem-itere) | Infusion | Oral | SI, SO | AT46 |
|  |  |  |  | Toothache | Crushed | Hold with teeth |  |  |
| *Croton macrostachyus* Del. [Euphorbiaceae] | Mekenisa (G), Wanshehena (G) | T | L  (bud) | Wound, Blood clotting, Tinea versicolor (Bechero), Common wart | Crushed | Topical | All sites | AT53 |
|  |  |  |  | Nasal congestion | Extract the juice/ squeezed | Nose drops |  |  |
|  |  |  | B, L | Indigestion (Qiter), Abdominal pain (and bloating, Intestinal parasite), Retained placenta | Infusion/Decoction | Oral |  |  |
|  |  |  | L | General malaise (Michi), Headache | Decoction, boiled, extract the juice/ squeezed | Oral, Steam bath, Nose drops |  |  |
|  |  |  |  | Jaundice | Infusion | Oral |  |  |
|  |  |  |  | Swelling | Boiled | Steam bath |  |  |
|  |  |  | Se | Pyoderma (Wegfiy, Kofa) | Crushed | Topical |  |  |
| *Euphorbia abyssinica* Gmel. [Euphorbiaceae] * | Qel-qal (G) | S | Lat | Piles | Extract the latex | Topical | W | AT78 |
| *Euphorbia cotinifolia* L. [Euphorbiaceae] * |  | S | Lat | Tinea versicolor (Bechero) | Extract the latex | Topical | ME | AT79 |
| *Euphorbia schimperiana* Scheele [Euphorbiaceae] | Edemo (G), Aybe-gedemo (Mq, Si) | H | Lat | Common warts “Qintebiye (G)” | Extract the latex | Topical | C, W, MQ, MA, SI, SO | AT80 |
|  |  |  | R | Indigestion (Qiter) | Infusion | Oral |  |  |
| *Euphorbia tirucalli* L. [Euphorbiaceae] |  | S | Lat | Piles, Common warts (Qintebiye (G)), Dandruff | Extract the latex | Topical | C, Q, W, MQ, ME, MA, SI, SO | AT81 |
| *Jatropha curcas* L. [Euphorbiaceae]* | Qondali (G) | S | Se | Epilepsy | Infusion | Oral | MQ | AT104 |
| *Ricinus communis* L. [Euphorbiaceae] | Gulo (G) | S | Se | Chigger bites (Mujelia, Ferfer) | Infusion  With butter | Topical | W, MQ, MA | AT169 |
|  |  |  | L, Se | Malaria, intestinal parasite | Infusion  With milk | Oral |  |  |
| *Acacia abyssinica* Hochst. ex Benth. [Fabaceae] | Teme-gerar (Q) | T | B | Indigestion (Qiter) | Decoction | Oral | W, ME, SI | AT02 |
|  |  |  | L | Malaria | Infusion | Oral |  |  |
|  |  |  |  | Tonsillitis | Infusion | Gargle to rinse the throat |  |  |
|  |  |  |  | Wound | Extract the juice/ squeezed | Topical |  |  |
| *Acacia seyal* Del. [Fabaceae] | Wacho-gerar (G, M), Urbu (G) | T | B | Indigestion (Qiter),  Liver complaint (Qoya, Seme dinku) | Decoction | Oral | W, MQ, ME, MA, SI, SO | AT03 |
|  |  |  |  | Tonsilitis | Infusion | Gargle to rinse the throat |  |  |
|  |  |  | St | Tinea versicolor (Bechero) | Collect water from wet burning stem | Topical |  |  |
| *Albizia schimperiana* Oliv. [Fabaceae] | Sasa (A) | T | Rb | Skin burn | Crushed | Topical | ME, SO | AT11 |
| *Caesalpinia decapetala* (Roth) Alston [Fabaceae] | Qeretef (A, G) | S | L  (bud) | Toothache | Crushed/chewed | Hold with teeth | SI | AT30 |
| *Calpurnia aurea* (Ait.) Benth. [Fabaceae] | Zegnet, Singo (G) Ticho (S) | S | L, St | Toothache | Crushed | Hold with teeth | C, Q, W, MQ, MA, SI, SO | AT31 |
| *Crotalaria incana* L. [Fabaceae] | Meza qiter (G)  Yejeb ater(A) | H | L | Wound, Skin burn | Crushed | Topical | ME | AT52 |
| *Lens culinaris* Medik. [Fabaceae]* |  | H | Se | Herpes zoster | Crushed | Topical | Q, ME, SI | AT115 |
| *Pseudarthria hookeri* Wight & Arn. [Fabaceae] |  | H | R | Liver complaint (Qoya, Seme dink | Crushed/Pounded | Smell | Q | AT159 |
| *Rhynchosia minima* (L.) DC. [Fabaceae] | Yefur enzir (G) | H | L | herpes zoster (- wound on nose which expands in time) | Crushed | Topical | SO | AT168 |
| *Senna multiglandulosa* (Jacq.) Irwin & Bameby [Fabaceae] |  | S | Se | Gonorrhoea (Emate) | Infusion | Oral | SO | AT182 |
| *Senna* *septemtrionalis* (Viv.) Irwin & Bameby [Fabaceae] | Chachate (A), Sememeki (G) | S | L | Wound, Pyoderma “Wegfiy, Kofa”, “Silensa”, Dandruff, Common warts, Snake bite, Antrax | Crushed  -With butter | Topical | ME, MA, SI, SO | AT183 |
| *Dovyalis* *abyssinica* (A. Rich.) Warb. [Flacourtiaceae] | Koshim (A) | S | L, B | Indigestion (Qiter) | Infusion/Decoction | Oral | C, Q | AT68 |
| Geran*i*um *arabicum* Forssk. [Geraniaceae] |  | H | L | Wound | Extract the juice | Topical | ME | AT88 |
| *Hydnora* *johannis* Becc. [Hydnoraceae] | Dechemerech (MQ) | H  /Epiphyte | Whole  part | Localized swelling | Crushed/powdered  With butter | Topical | SI, MQ | AT98 |
| *Apodytes dimidiata* E. Mey. ex Arn. [Icacinaceae] | Wendemu (A), Gefye (G) | T | B | Diarrheal disease (Child) (Ye-dengiya-qar or Ye-sequriye (Gu), cholera,  General health (increase weight, improve strength and boost immunity of infants) | Decoction | Oral | C, Q, MQ, ME, SO | AT16 |
| *Gladiolous* *abyssinicus* (Brongn. ex Lemaire) Goldblatt & de Vos [Iridaceae] | Inzerezyi (G) | H | Cr | Toothache, Anthrax (Shem-itere) | Crushed/chewed | Hold with teeth | C, Q | AT89 |
| *Ajuga integrifolia* Buch-Ham. [Lamiaceae] * | Anamuro, ema telit (G), Anamurcho (Q) | H | L | Anorexia, Rheumatic pain (Deme-tukiy), Abdominal pain | Infusion/ Decoction | Oral | All sites | AT10 |
| *Becium obovatum* (E. Mey. ex Benth.) N.E. Br. [Lamiaceae] |  | H | R | Anthrax (Shem-itere) | Infusion | Oral | Q | AT23 |
| *Clerodendrum myricoides* (Hochst.) Vatke [Lamiaceae] | Aleg (A), Hanigo (G, Q) | H | L | Abdominal pain, Diarrheal disease (Child) (Ye-dengiya-qar or Ye-sequriye (Gu), Tonsillitis | Infusion | Oral | All sites | AT45 |
|  |  |  |  | Evil spirit (Dorer, Likift, Buda) | Crushed, Infusion | Bath, oral |  |  |
| *Fuerstia africana* T.C.E. Fr. [Lamiaceae] | Yegiye ensosla (G), Nazoli (S), Hureda (M) | H | L | General malaise (Mich), Headache | Infusion | Nose drops | W, MQ, ME, SI, SO | AT86 |
| *Leonotis ocymifolia* (Burm. f.) Iwarsson [Lamiaceae] | Chenbolibi (S) | S | L | Ascariasis | Infusion | Oral | MA, SI | AT116 |
| *Leucas argentea* Gurke [Lamiaceae] | Fiza, Kiza (G) | H | L | Indigestion (Qiter), Diarrhea, Abdominal pain, Constipation (Children) | Infusion/Decoction  -With milk | Oral | C, W, ME, MA, SI | AT118 |
| *Ocimum basilicum* L. [Lamiaceae]* | Meso bela (A) | H | L | Abdominal bloating | Infusion | Oral | ME, MA | AT135 |
| *Ocimum lamiifolium* Hochst. ex Benth. [Lamiaceae]* | Damakese (A) | S | L | Common cold, coughing, Pneumonia (Sinbabie), general malaise(Michi), Headache | Decoction,  With coffee, Extract the juice/squeeze | Oral, Bath, Nose drops | All sites | AT136 |
|  |  |  |  | Amoebiasis; Abdominal pain, Abdominal bloating, | Decoction | Oral |  |  |
|  |  |  |  | Toothache | Crushed | Hold with teeth |  |  |
| *Ocimum urticifolium* Roth [Lamiaceae] | Yelebe fuanfa, Delibekera (Q) | S | L | Toothache | Crushed | Hold with teeth | W, MQ, ME, SI | AT137 |
|  |  |  |  | General malaise (Michi), Common cold | Decoction, boiled | Oral, steam bath |  |  |
| *Otostegia tomentosa* A. Rich. [Lamiaceae] | Yesetan abeba (A) | S | L | Wound, Pyoderma (Wegfiy, Kofa) | Crushed | Topical | MA | AT140 |
| *Plectranthus cylindraceus* Hochst. ex Benth. [Lamiaceae] | Qintele sat (G) | H | L | Swelling | Crushed | Topical | SO | AT152 |
|  |  |  |  | Evil spirit (Dorer, Likift, Buda), Rheumatism | Crushed | Bath |  |  |
| *Plectranthus edulis* (Vatke) Agnew [Lamiaceae]* | Yegurage denicha (A) | H | L | Malaria | Decoction | Oral | C | AT153 |
| *Plectranthus* cf. *minutiflorus* Ryding [Lamiaceae] | Aomar (S) | H | Arial part | Retained placenta | Decoction | Oral | W | AT154 |
| *Premna schimperi* Engl. [Lamiaceae] | Teqoqe (G), Wankisa (G, Q), Ye fiyel kolo (A) | S | L | Toothache | Crushed | Hold with teeth | C, Q, W, SI | AT157 |
| ***Pycnostachys abyssinica*** Fresen. [Lamiaceae] | Fuanfa (G) | S | L | Abdominal pain, Diarrhea, Malaria, General malaise (Michi), General health (increase weight, improve strength and boost immunity of infants) | Infusion/Decoction | Oral | C, W, MQ, ME, SI | AT162 |
|  |  |  | L | Eye infection | Cut small pieces | Topical |  |  |
| *Rosmarinus officinalis* L. [Lamiaceae] * |  | S | L | Hypertension | Infusion/Decoction  -with coffee | Oral | MA, SO | AT171 |
| *Salvia nilotica* Jacq. [Lamiaceae] | Amam, Guneliye, Meza qutel (G) | H | L | Wound, Pyoderma “Wegfiy, Kofa”, Skin burn, Eye infection “wucher, Meza” | Crushed | Topical | C, W, ME, MA, SI, SO | AT177 |
|  |  |  |  | General malaise (Michi) | Infusion/Decoction, boiled | Oral, Steam bath |  |  |
| *Satureja abyssinica* (Benth.) Briq. [Lamiaceae] | Debeqqo (G) | H | L, St | Indigestion (Qiter), Abdominal pain, Abdominal bloating | Infusion | Oral | MQ, ME, MA, SI, SO | AT178 |
| *Satureja punctata* (Benth.) Briq. [Lamiaceae] | Debeqqo (G) | H | L | Indigestion (Qiter) | Infusion | Oral | C, ME, SO | AT179 |
| ***Thymus schimperi*** Ronniger [Lamiaceae] | Tosign (A) | H | L | Hypertension | Decoction | Oral | C, MA, SI | AT201 |
| *Persea americana* Mill. [Lauraceae]* | Abokato (A) | T | Fr | Dandruff | Crushed | Topical | Q, MQ, ME, SI | AT145 |
|  |  |  | L | Anemia | Decoction | Oral |  |  |
| *Linum usitatissimum* L. [Linaceae]* | Telba (A) | H | Se | Retained placenta, Amoebiasis, Abdominal pain, Gastritis, Constipation | Infusion/Decoction | Oral | All sites | AT119 |
| *Buddleja polystachya* Fresen. [Loganiaceae] | Anfar (A, G) | S | R | Gonorrhea (Emate) | Infusion | Oral | C, SO | AT29 |
|  |  |  | L | Tonsillitis | Infusion | Oral |  |  |
| ***Phragmanthera macrosolen*** (A. Rich.) M. Gilbert [Loranthaceae] | Teqetla (A) (hemi-parasite growing on *Acacia* sp*.*) | S | Whole part | Evil spirit (Dorer, Likift, Buda), General maliasis (mich) | Infusion, Boiled | Oral, Steam bath | MA, SO |  |
| *Tapinanthus globiferus* (A. Rich.) Tieghem [Loranthaceae] | Teqetla (A) (hemi-parasite growing on coffee, chat, peach) | S | Whole part | Evil spirit (Dorer, Likift, Buda), Depression, General malaise (Michi) | Burn, Boiled | Smell the smoke, Steam bath | ME, MA, SI, SO | AT198 |
|  |  |  |  | Retained placenta | Infusion | Oral |  |  |
| *Caucanthus auriculatus* Forssk. [Malpighiaceae] |  | C | L | Epilipsy (Azurit) | Infusion | Oral | C, MQ | AT37 |
| *Abutilon* sp. [Malvaceae] | Borer (S) | S | L | Headache | Extract the juice/squeeze | Nose drops | W | AT01 |
| *Hibiscus berberidifolius* A. Rich. [Malvaceae] | Yekesheshiye (G) | S | R | Amoebiasis | Infusion | Oral | MA, SI | AT97 |
|  |  |  | L | Wound ( Silensa); Herpes zoster; Skin burn | Crushed | Topical |  |  |
| *Hibiscus micranthus* L. f. [Malvaceae] | Badefacha (S, M) | H | L | Indigestion (Qiter) (dyspepsia) | Infusion | Oral | MA, SI | AT99 |
| *Malva verticillata* L. [Malvaceae] | Lite (A) | H | R | Intestinal parasites | Infusion | Oral | SI | AT124 |
| *Pavonia urens* Cav. [Malvaceae] | Menatef (A, G) | H | L, R | Indigestion (Qiter), Diarrhea (Children), Abdominal bloating, Excess vomiting (Hyperemesis) | Infusion | Oral | ME, MA, SO | AT143 |
| *Sida rhombifolia* L. [Malvaceae] | \| Badefacha (S, M) \| Root \| Diarrhea, amebiasis \| \| --- \| --- \| --- \| | H | R | Abdominal pain, Diarrhea, amebiasis | Infusion | Oral | C | AT184 |
| *Sida schimperiana* Hochst. ex A. Rich. [Malvaceae] | Chifereg (A), Anjajewet (G) | S | R | Teniasis “Chima (G)”, “Seto (K)”, Diarrhea, General malaises | Infusion | Oral | MQ, ME, SO | AT185 |
| *Dissotis senegambiensis* (Guill. & Perr.) Triana [Melastomataceae] |  | H | L | Pyoderma (Wegfiy, Kofa), Swelling | Crushed | Topical | MA | AT67 |
| *Ekebergia capensis* Sparrm. [Meliaceae] | Wulel (S), Guareba (G) | T | L | Toothache | Crush and Boil | Hold with teeth | C, ME, MA | AT71 |
|  |  |  | L, Se | Wound (bitten by hyena) | Crushed | Topical |  |  |
| *Melia azedarach* L. [Meliaceae] |  | T | L | Malaria | Infusion | Oral | Q, MQ, ME | AT128 |
|  |  |  |  | Hypertension | Decoction | Oral |  |  |
| *Bersama abyssinica* Fresen. [Melianthaceae] | Kurata, Hureta (G) | T | Se | Hemorrhoid, Skin burn, Dandruff, Scabies | Crushed | Topical | C, Q, MA | AT24 |
| *Stephania abyssinica* (Dillon & A.Rich.) Walp. [Menispermaceae] | Foreformat , Kelalla (G), Meqeres (S) | H | R | Indigestion (Qiter), Stabbing pain, Liver complaint (Qoya, Seme dinku), Malaria, Diarrhea, General malaise (Mich), | Infusion | Oral | C, MQ, ME, MA, SI, SO | AT195 |
| *Ficus sur* Forssk.  [Moraceae] | Sobial, Neche-warka (G) | T | Fr | Pyoderma (Wegfiy, Kofa) | Extract the juice | Topical | ME | AT82 |
| *Ficus sycomorus* L. [Moraceae] | Wedisha (G) | T | Lat | Pyoderma “Wegfiy, Kofa” | Extract the latex | Topical | C, ME, SI | AT83 |
| *Ficus vasta* Forssk. [Moraceae] | Azodichito (Q), Shebra, Werha (G), Neche-shola, Hufonda (S) | T | B | Indigestion (Qiter) | Infusion | Oral | Q, W | AT84 |
| *Moringa stenopetala* (Bak. f.) Cuf. [Moringaceae]* | Shiferaw (A) | T | L | Malaria, Gastritis, Hypertension | Decoction | Oral | Q, MQ | AT131 |
| *Ensete ventricosum* (Welw.) Cheesman [Musaceae] * | Eset (G, S), Enset (A) | S | Cr | Liver complaint (Qoya, Seme dinku), Bone fracture, Retained placenta, Indigestion (Qiter), Toothache | Cooked | Oral | All sites |  |
|  |  |  | St |  | Extract watery juice from pseudo-stem | oral |  |  |
| *Myrica salicifolia* A. Rich. [Myricaceae] | Cheta, telota (G) | T | B | Indigestion (Qiter) | Infusion/ Decoction | Oral | MA | AT132 |
| *Embelia schimperi* Vatke [Myrsinaceae] | Enqueqwe (G), Enqoqo (A) | C | Se | Teniasis “Chima (G)”, “Seto (K)” | Infusion | Oral | Q, ME | AT73 |
| *Maesa lanceolata* Forssk. [Myrsinaceae] | Aguaj (G), Qelew (A) | T | L | Malaria, Intestinal parasites | Infusion | Oral | ME, SO | AT123 |
| *Myrsine africana* L. [Myrsinaceae] | Qechemewe (G), Qechemo (A) | S | Se | Abdominal pain ( also as prevention) | Infusion  -Mixed with ‘TEEF’ and baked | Oral | ME, MA, SO | AT133 |
| *Eucalyptus globulus* Labill. [Myrtaceae] * | Antakirt (G) | T | L | Common cold, Headache, General malaise “Mich” | Boiled | Steam bath | C, Q, ME, MA, SI, SO | AT76 |
| *Psidium guajava* L. [Myrtaceae]* | Zeyetun (A) | T | Ex | Wound, Dandruff | Crushed | Topical | Q, ME, MA | AT160 |
|  |  |  | Fr | Constipation | Crushed | Oral |  |  |
|  |  |  | L | Hemorrhoid | Boiled | Wash |  |  |
| *Jasminum abyssinicum* Hochst. ex Dc. [Oleaceae] | Torso (G) | C | L | Eye disease | Extract the juice/ squeeze | Topical | ME | AT103 |
|  |  |  | R | Wound | Extract the juice | Topical |  |  |
| *Olea europaea* L. subsp. *cuspidata* (Wall. ex G.Don [Oleaceae]* | Wera (A), Bunne (G) | T | St, L | Toothache | Crushed (warm)/Chewed | Hold with teeth | All sites | AT138 |
|  |  |  | L | Indigestion (Qiter) | Infusion/Decoction | Oral |  |  |
| *Olinia rochetiana* A. Juss. [Oliniaceae] | Tife (A), Shumelo (S) | T | B, L, St | Wound, Pyoderma (Wegfiy, Kofa) | Crushed | Topical | MA, SI | AT139 |
|  |  |  |  | Toothache | Crushed/chewed | Hold with teeth |  |  |
| *Oxalis corniculata* L. [Oxalidaceae] | Yetay asebo (G) | H | Whole part | Abdominal pain | Decoction | Oral | Q, SO | AT141 |
|  |  |  |  | Swelling | Crushed | Topical |  |  |
| *Argemone mexicana* L.  [Papaveraceae] | Nech-lebash (A) | H | Lat | Wound, Dandruff | Extract the latex | Topical | MQ, SI | AT17 |
| *Phytolacca dodecandra* L’Herit. [Phytolacaceae] | Endod (A) | S | R | Rabies | Infusion | Oral | C, W, ME, SO | AT148 |
|  |  |  | L, R | Tonsillitis | Infusion | Gargle to rinse the throat |  |  |
| *Pittosporum viridiflorum* Sims [Pittosporaceae] | Hunbosho (Si), Ulaga (G) | T | L | Coughing, Pneumonia (Sinbabie), TB, Abdominal pain | Infusion | Oral | C, W, ME | AT149 |
| *Plantago lanceolata* L. [Plantaginaceae] | Yefur enzir, Qoshqoshye (G) | H | L | Wound | Crushed | Topical | W, MQ, MA, SI | AT150 |
|  |  |  |  | Tonsillitis | Infusion | Gargle to rinse the throat |  |  |
|  |  |  |  | General malaise (Michi) | Extract the juice/squeeze | Nose drops |  |  |
| *Plantago palmata* Hook.f. [Plantaginaceae] |  | H | L | Wound | Crushed | Topical | C, MA | AT151 |
| *Arundinaria alpina* K. Schum. [Poaceae] | Eneet, awsar(G) | S | L | Abdominal pain, Diarrheal disease (Child) (Ye-dengiya-qar or Ye-sequriye (Gu) | Infusion | Oral | C, MA | AT20 |
| *Cymbopogon citratus* (DC. ex Nees) Stapf [Poaceae]* | Hiticho (M), Deg sar (G, S), Moseret (G) | H | L | Anthrax (Shem-itere), Dengetegna | Infusion | Oral | MQ, MA, SO | AT57 |
|  |  |  | R, L | Abdominal pain | Infusion | Oral |  |  |
| *Dactyloctenium aegyptium* (L.) Willd. [Poaceae] |  | H | Se | Anthrax (Shem-itere) | Decoction  Baked with barely flour | Oral | MA | AT61 |
| *Eleusine floccifolia* (Forssk.) Spreng. [Poaceae] |  | H | L | Spider bite | Crushed | Topical | C, SO | AT72 |
| *Zea mays* L. [Poaceae] * |  | H | R | Indigestion (Qiter) | Infusion | Oral | MQ, MA, SI | AT212 |
| *Podocarpus falcatus* (Thunb) R. Br. Ex Mirb. [Podocarpaceae] | Zigba (A, G) | T | B, L | Coughing | Decoction | Oral | C, MA, SI | AT155 |
|  |  |  |  | Latex - wound |  |  |  |  |
| *Polygala sadebeckiana* Gurke [Polygalaceae] | Shime-itere chiza, Qiteriye (G), Felfel (A), Shime yeter zebo (Q) | H | R | Toothache | Crushed | Hold with teeth | C, Q, MA | AT156 |
|  |  |  |  | Anthrax (Shem-itere), Abdominal pain, Indigestion (Qiter) | Infusion | Oral |  |  |
| *Oxygonum sinuatum* (Meisn.) Dammer [Polygonaceae] | Sherinto (S) | H | L | Wound | Crushed | Topical | C | AT142 |
| *Persicaria senegalensis* (Meisn.) Sojak [Polygonaceae] | Hobet (S), Nech azhe (G) | H | L | Retained placenta | Infusion | Oral | C, W, MQ, SI | AT146 |
|  |  |  |  | Swelling esp. on neck “Kinta” | Crushed | Topical |  |  |
| *Rumex abyssinicus* Jacq. [Polygonaceae] | Hambo (G), Yebech- ambo, Weshe temo (Q) | H | R | Gonorrhoea (Emate), | Infusion/Decoction | Oral | Q, W, MA, SI | AT173 |
|  |  |  | R | Liver complaint (Qoya, Seme dinku), Kidney problem | Infusion/Decoction | Oral |  |  |
| *Rumex nepalensis* Spreng. [Polygonaceae] | Chabe (Q), Tumeya, tuya, yegrid amber(G) | H | R | Abdominal pain, Abdominal bloating, Diarrhea (Children), Indigestion (Qiter) | Infusion, Crushed/Chewed | Oral | All sites | AT174 |
|  |  |  | R | Wound, Dandruff (with sores on scalp) | Crushed | Topical |  |  |
|  |  |  | R, L | Tonsillitis | Extract the juice | Gargle to rinse the throat |  |  |
| *Rumex nervosus* Vahl [Polygonaceae] | Angago, yegeye chima (G) | S | L (bud) | Herpes zoster, common wart, Tinea versicolor (Bechero) | Crushed | Topical | C, ME | AT175 |
| *Lysimachia ruhmeriana* Vatke [Primulaceae] |  | H | L | Evil spirit (Dorer, Likift, Buda) | Infusion | Oral | Q | AT122 |
| *Punica granatum* L. [Punicaceae]* | Roman (A) | S | Fr | Excess vomiting (Hyperemesis) | Crushed | Oral (eaten) | SI | AT161 |
| *Clematis longicauda* Steud. ex A. Rich. [Ranunculaceae] |  | C | L | General malaise (Mich) | Infusion | Nose drops | C, MA | AT43 |
|  |  |  |  | Hemorrhoid | Crushed | Topical |  |  |
| *Clematis simensis* Fresen. [Ranunculaceae] | Fida (A), Yegawa wedero; Sem che cheniyit; Aze, Hanz'o (G) | C | L | Pyoderma (Wegfiy, Kofa), Wound | Crushed | Topical | Q, W, MQ, ME, SI, SO | AT44 |
|  |  |  |  | Tonsillitis | Infusion | Gargle to rinse the throat |  |  |
|  |  |  |  | Eye infection (Wucher) | Extract the juice/ squeezed | Nose drops |  |  |
| *Nigella sativa* L. [Ranunculaceae] * | Tiqure azemud (A), Gmebel menzuta (Q) | H | Se | Abdominal pain, General malaise | Infusion  -Honey | Oral | C, Q, W, MQ, SI | AT134 |
|  |  |  | Se | Headache; | Grind and soak in water/ Infusion  -With butter | Nose drops |  |  |
| *Ranunculus multifidus* Forssk. [Ranunculaceae] |  | H | R | Cancer, Ulcer, ‘Neqersa’, Pyoderma ‘Wegfiy, Kofa’ | Infusion | Oral | ME | AT163 |
| *Rhamnus prinoides* L. Herit. [Rhamnaceae]* |  | S | L (bud) | Tonsilitis | Infusion  -With butter | Gargle to rinse the throat | ME, SO | AT164 |
| *Hagenia abyssinica* (Bruce) J.F. Gmel. [Rosaceae] |  | T | Fl | Teniasis “Chima (G)”, “Seto (K)”, Abdominal pain, Diarrhea | Infusion | Oral | C, Q, W, ME, MA, SI, SO | AT93 |
|  |  |  | Fl, Se | Anorexia, Indigestion (Qiter), Malaria | Infusion | Oral |  |  |
| *Prunus persica* (L.) Batsch [Rosaceae]* | Kok (A, G) | T | L | General malaise “Michi”, Indigestion (Qiter), Stabbing pain, Anthrax (Shem-itere) | Infusion | Oral | C, MA | AT158 |
| *Rosa hybrida* L. [Rosaceae]* | Mahle-weld () | S | Fl | Eye infection (Wucher) | Infusion | Drop into the eyes | C, Q, ME | AT170 |
| *Coffea arabica* L. [Rubiaceae]* |  | S | Se | Wound, Skin burn | Roast and grind | Topical | C, Q, MQ, ME, SI | AT47 |
|  |  |  | L | General health (increase weight, improve strength and boost immunity of infants) | Decoction | Oral |  |  |
| *Gardenia ternifolia* Schumach. & Thonn. [Rubiaceae]* | Genbalyi (A), Habuliy (G) | T | L | Malaria | Decoction | Oral | Q | AT87 |
| *Pentas schimperiana* (A. Rich.) Vatke [Rubiaceae] | Mesabur (Q) | S | L, R | Back pain, Bone fracture | Infusion/ Decoction | Oral | C, ME | AT144 |
| *Rubia cordifolia* L. [Rubiaceae] | Enchiber (G) | H | R | Toothache | Crushed | Hold with teeth | C, MA, SO | AT172 |
|  |  |  | L | Toothache | Crushed | Hold with teeth |  |  |
|  |  |  | L | Evil sprit (Dorer) | Infusion/ Extract the juice | Oral |  |  |
|  |  |  | R | Swelling, Snake bite, | Crushed | Bath |  |  |
|  |  |  | R | Jaundice | Infusion | Oral |  |  |
|  |  |  |  |  |  |  |  |  |
| *Citrus aurantifolia* (Christm.) Swingle [Rutaceae]* | Lomi (A) | T | Fr | Tonsillitis; Abdominal pain, Intestinal parasites; | Infusion | Oral | C, Q, W, MQ, ME, MA, SI, SO | AT39 |
|  |  |  | L | Anthrax (Shem-itere) | Infusion | Oral |  |  |
|  |  |  | Fr | Wound, Tinea versicolor (Bechero), bad foot smell | Infusion | Topical |  |  |
|  |  |  | Fr | Toothache | Extract the juice/ squeezed | Apply on teeth |  |  |
| *Citrus aurantium* L.  [Rutaceae]* | Hometate (A) | T | Fr | Hyperemesis | Infusion | Oral | C, W, ME, Sl | AT40 |
| *Citrus medica* L.  [Rutaceae]* | Terengo (A) | S | Ex, Fr | Evil spirit (Dorer, Likift, Buda) | Infusion | Oral | Q, MA | AT41 |
| *Clausena anisata* (Willd.) Benth. [Rutaceae] | Lemuche (A) | T | L | General malaise (Michi), Stabbing pain, Malaria, Evil spirit (Dorer, Likift, Buda) | Infusion | Oral | C, MA, ME, SO | AT42 |
|  |  |  |  |  | Boiled | Steam bath |  |  |
| *Ruta chalepensis* L. [Rutaceae]* | Tena adam | H | L, Se | Abdominal pain, Diarrhea, Abdominal bloating, General malaise(Michi), Evil spirit (Dorer, Likift, Buda), Rheumatic Pain, Afterpains, Common cold, Coughing, Headache, Stabbing pain, Loss of appetite (Anorexia ), smelly flatus | Infusion  -With coffee/tea | Oral | C, Q, W, MQ, ME, MA, SI, SO | AT176 |
| ***Teclea nobilis*** Del. [Rutaceae] | Ader (G) | T | L | Indigestion (Qiter), Abdominal pain | Infusion | Oral | MA | AT199 |
| *Toddalia asiatica* (L.) Lam. [Rutaceae] | Asegomare, Biter, Zega berbero (G) | C | L | Teniasis “Chima (G)”, “Seto (Q)” | Infusion | Oral | ME, MA | AT202 |
|  |  |  | L | Rheumatic pain | Crushed (Cold/warm) | Topical |  |  |
| *Scolopia theifolia* Gilg [Salicaceae] | Koshim (A), Aweta (G) | S | St | Stop weird pregnancy craving | Crushed | Oral | MA | AT181 |
| *Sideroxylon oxyacanthum* Baill. [Sapotaceae] | Miteja (G) | S | St, B | Indigestion (Qiter) | Infusion/ decoction | Oral | C, ME, MA | AT186 |
| *Verbascum sinaiticum* Benth. [Scrophulariaceae] | Yemar enzir (G), Halemecha, Huleten huta (M), Yumar amel ( Q, S) | H | Rb, R | Abdominal pain, Diarrhea, Diarrheal disease (Child) (Ye-dengiya-qar or Ye-sequriye (Gu), cholera , General health (increase weight, improve strength and boost immunity of infants), Indigestion (Qiter) | Infusion/ Decoction | Oral | C, W, MQ, ME, MA, SI, SO | AT203 |
|  |  |  | L | Liver complaint (Qoya, Seme dinku), Indigestion (Qiter) | Infusion/Decoction | Oral |  |  |
|  |  |  |  | Evil spirit (Dorer, Likift, Buda) | Crushed | Bath |  |  |
| *Brucea antidysenterica* J.F .Mill. [Simaroubaceae] | Yemoyet bosha (G) | T | L | Evil spirit (Dorer, Likift, Buda) | Crushed | Topical /holding | ME, MA | AT28 |
|  |  |  | Se | Wound (on male genital part) | Roast and grind | Topical |  |  |
| *Smilax aspera* L. [Smilacaceae] | Yezogare gaje(G) | C | L | Liver complaint (Qoya, Seme dinku) | Infusion | Oral | ME | AT188 |
| *Capsicum annuum* L. [Solanaceae]* |  | H | Fr | Toothache | Crushed | Hold with teeth |  |  |
| *Datura stramonium* L. [Solanaceae] | Mechara (M, S), Azaza (G, Q) | H | Se | Toothache | Mix with butter and burn | Inhale the smoke direct into the teeth using tube | All sites | AT63 |
|  |  |  | Se, L | Dandruff, Pyroderma (Wegfiy, Kofa), Headache, Hemorrhoid | Crushed  -With butter | Topical |  |  |
|  |  |  | L | Inflammation (Insect bite) | Extract the juice/squeezed | Topical |  |  |
| *Discopodium penninervium* Hochst. [Solanaceae] | Enchochika (G) | T | L (Old/  yellow) | Indigestion (Qiter), Abdominal pain (Children) | Infusion/Decoction | Oral | C, ME, SO | AT65 |
|  |  |  | L | General health (increase weight, improve strength and boost immunity of infants) | Infusion | Oral |  |  |
| *Lycopersicon esculentum* Mill. [Solanaceae]* |  | H | L | Urinary retention | Decoction | Oral | SO | AT121 |
| *Solanum anguivi Lam.* [Solanaceae] |  | S | Fr | Rabies, Snake bite | Infusion/cooked | Oral | SO | AT191 |
| *Solanum giganteum* Jacq. [Solanaceae] |  | S | L | Common warts, Anthrax (Shem-itere) | Crushed | Topical | Q, MA | AT192 |
| *Solanum incanum* L. [Solanaceae] | Embuay (A), Zereche (G), Yaqom-zaro (S) | S | R | Abdominal pain, Diarrhea, Indigestion (Qiter), General malaises (Michi) | Infusion /Chewed | Oral | C, Q, W, MQ, ME, MA, SI, SO | AT193 |
|  |  |  | Fr | Anthrax (Shem-itere) , Swelling | Crushed | Topical |  |  |
|  |  |  | Fr | Dandruff, Wound | Crushed | Topical |  |  |
|  |  |  | Fr, R | Tonsillitis | Infusion | Gargle to rinse the throat |  |  |
|  |  |  | Leaf | Epistaxis | Extract the juice/squeeze | Nose drops, Smell |  |  |
| *Solanum nigrum* L. [Solanaceae] | Emberebuniye(G) | H | Fr | Rabies | Infusion  -Mixed with ’TEEF’ and baked | Oral | C | AT194 |
|  |  |  | L | Wound, Dandruff (Sores on the scalp), common warts | Crushed | Topical |  |  |
| *Withania somnifera* (L.) Dunal [Solanaceae]* | Gezawa (A) | S | L, R | Evil spirit (Dorer, Likift, Buda), General malaise (Michi), Swelling, Itching | Infusion, Decoction, Boil, Burn | Oral, Steam bath, Smoke | All sites | AT210 |
|  |  |  |  | Abdominal pain, Diarrheal disease (Child) (Ye-dengiya-qar or Ye-sequriye (G), cholera | Infusion | Oral |  |  |
| *Gnidia stenophylla* Gilg [Thymelaceae] | Mesemes (G) | H | L, R | Indigestion (Qiter), Abdominal pain, Diarrhea, Retained placenta, Rabies, Gonorrhoea (Emate, Chebeto) | Infusion | Oral | C, SO | AT90 |
| *Lantana trifolia* L. [Verbenaceae] | Yabohun qolo (G) | S | L | Headache | Infusion | Nose drops | W | AT114 |
|  |  |  |  | Common cold | Infusion | Oral |  |  |
|  |  |  | Se | Toothache | Crushed | Hold with teeth |  |  |
| ***Lippia adoensis* Hochst. ex Walp. var. *adoensis*** [Verbenaceae] | Kesse (A) | S | L | Abdominal pain, Diarrhea, Indigestion (Qiter), General malaise (Michi) | Infusion | Oral | W, MQ, SI, SO | AT120 |
|  |  |  |  | Toothache | Crushed | Hold with teeth |  |  |
| *Verbena officinalis* L. [Verbenaceae] | Qesqes (G) | H | L, R | Abdominal pain, Diarrhea, Indigestion (Qiter), Malaria, Excess vomiting (Hyperemesis), General malise (Michi), | Infusion  -With milk, whey | Oral | All sites | AT204 |
| *Cyphostemma cyphopetalum* (Fresen.) Desc. Ex Wild & R.B. Dr umm [Vitaceae] | Toleje (G) | C | L | Wound (Silensa, on neck) | Crushed | Topical | ME, MA, SI | AT59 |
| *Cyphostemma niveum* (Hochst. Ex Schweinf.) Desc. [Vitaceae] |  | C | L | General malaise (Mich) | Boiled, Extract the juice/ squeezed | Steam bath, Nose drops | ME, MA, SI | AT60 |
|  |  |  | St | Swelling, Skin burn | Crushed | Topical |  |  |
| *Rhoicissus tridentata* (L. f.) Wild & Drummond [Vitaceae] | Lalota, Yegawa wedero (G), Dubi fizuta (Q) | C | L | Liver complaint (Qoya, Seme dinku) | Infusion | Oral | W | AT165 |
| *Zingiber officinale* Roscoe [Zingiberaceae] * |  | H | Rh | Tonsillitis, Abdominal pain, Toothache, Common cold, Coughing | Decoction, crushed/chewed | Oral | All sites | AT213 |
| *Aframomum corrorima* (Braun) P.C.M. Jansen)  [Zingiberaceeae] * | Wekashe (G) | H | Se | Tonsillitis | Infusion | Gargle to rinse the throat | SI | AT07 |
| *Calvatia* sp. [Agaricaceae] | Seme dinku | Fungus | Whole part | Liver complaints | Decoction | Oral | SI, W |  |
