# Supplementary material for: Medicinal plant use practice in four ethnic communities (Gurage, Mareqo, Qebena, and Silti), south central Ethiopia
Source: J Ethnobiol Ethnomed. 2020 May 24;16:27. doi: 10.1186/s13002-020-00377-1 (PMC7245860; doi:10.1186/s13002-020-00377-1)
Supplement: Supplementary file 2 — Additional file 2. List of medicinal plants used to treat livestock ailments: scientific name, family; vernacular name; growth form; plant parts used; ailment treated; methods of preparation; routes of administration; study sites; voucher number [file 13002_2020_377_MOESM2_ESM.docx]

Additional file 2. List of medicinal plants used to treat livestock ailments: scientific name, family; vernacular name; growth form; plant parts used; ailment treated; methods of preparation; routes of administration; study sites; voucher number

**Key**: Vernacular name-Guragegna (G); Qebena (Q); Mareqo (M); Siltigna (S); Amharic (A). Growth form (GF)- Tree (T); Shrub (S); Herb (H); Climber (C), Epiphyte. Part used (PU) -Leaf, L; Root, R; Fruit, Fr; Exocarp, Ex; Bark, B; Stem, St; Root bark, Rb; Flower, Fl; Bulb, Bu; Seed, Se; Corm, C; Tuber, Tu; Rhizome, Rh; Latex, Lat. Methods of preparation (MOP): Extract the Juice- Squeezed with little water added; Crushed – plant part material pounded/powdered; Infusion- soaked in water and filter; Decoction-boiled in water and filtered. Routes of administration (ROA). Study sites-Cheha (C); Qebena (Q); Wulbareg (W); Mareqo (MQ); Meskan (ME); Muhir-Aklil (MA); Silti (SI); Sodo(So).

| **Scientific name [Family]** | **Vernacular name** | **GF** | **PU** | **Traditional use** | **MOP** | **MOA** | **Study sites** | **Voucher No.** |
| --- | --- | --- | --- | --- | --- | --- | --- | --- |
| *Hypoestes forskaolii* (Vahl) R. Br. [Acanthaceae] | Yete beder (G) | H | L | Increase milk production | Infusion | Oral | C, Q, MQ, ME, SI, SO | AT100 |
| *Acanthus eminens* C. B. Clarke [Acanthaceae] | Koshkoshe (A) | S | L | Wound, Sudden illness (Dengetegan) | Crushed, Infusion | Topical, Oral | C, MA, SO | AT214 |
| *Thunbergia ruspolii* Lindau [Acanthaceae] | Yangacha qomet, Afuakiyi (G) | H | L | Fattening (calf) | Crushed | Oral | C, MA, SO | AT200 |
| *Justicia schimperiana* (Hochst. ex Nees) T. Anders. [Acanthaceae] | Abegafuye, Hneba (G), Temuga (S, M | S | L | Sick livestock with erect and stiff hair, don't chew (Demuga); Newcastle disease (Fengel) | Infusion | Oral | All sites | AT106 |
| *Agave sisalana* Perrine ex Engl. [Agavaceae] | Alage (A) | S | L, R | Indigestion, Sudden illness (Dengetegna) | Infusion | Oral | ME, SI, W, SO | AT08 |
|  |  |  | L | Chicken mites | Infusion | Topical |  |  |
| *Aloe pubescens* Reyonolds. [Aloaceae] | Merdedeye, Yefuga gedel (G), Werab kershi (S) | H | L | Wound | Crushed (warm) | Topical | All sites | AT14 |
|  |  |  | Lat | Cough, Fattening | Crushed (warm) | Oral |  |  |
| *Aloe trichosantha* Berger [Aloaceae] | Merdedeye, Yefuga gedel (G), Werab kershi (S) | S | L | Swelling | Crushed (warm) | Topical | ME | AT215 |
| *Lannea schimperi* (A. Rich.) Engl. [Anacardiaceae] | Abariyet (G) | T | L | Bloat | Infusion | Oral | C | AT113 |
| *Schinus molle* L. [Anacardiaceae] | Treeman tree (A) | T | L | Bloat | Infusion | Oral | MQ, ME | AT180 |
|  |  |  |  | Repel house flies | Burn | Smoking for the house and keeping the piece |  |  |
| *Crinum* sp. [Amaryllidaceae] | Kuresh (G) | H | Tu | Anthrax, Blackleg, Trypanosomosis, Sudden illness (Dengetegan) | Infusion | Oral | C, MA | AT216 |
| *Foeniculum vulgare* Miller [Apiaceae] | Wet-anbo (G), Enshelal (Q), Aneshway (S), Ansho (M) | H | R, L | Bloat | Infusion | Oral | C, Q, W, MQ, ME, MA, SI, SO | AT85 |
| *Sauromatum venosum* (Ail.) Kunth [Araceae] | Nech-kuresh (G) | H | C | Anthrax (Shem-itere (G)) | Infusion | Oral | C, Q, MA | AT217 |
| *Phoenix reclinata* Jacq. [Arecaceae] | Zenebaba (A) | T | L | Eye infection (Wucher) | Infusion | Drop Into the eyes | C, ME, MA | AT218 |
| *Berkheya spekeana* Oliv. [Asteraceae] | Seheye, Enseheyet (G) | H | L | Retained placenta | Infusion | Oral | MA | AT219 |
| *Echinops hispidus* Fresen. [Asteraceae] | Yimar-soohe, Dender (G), umar soohe (Q), Haluta | H | R | Bloat, Fattening (Calf) | Infusion | Oral | SI, SO | AT69 |
| *Echinops kebericho* Mesfin. [Asteraceae] | Chosa (G), Kebericho (S, M, Q) | H | L, R | Diarrhea, Anthrax (Shem-itere (G)), Sudden illness (Dengetegan), prevent contagious disease | Infusion, burn | Oral, Smoked for the house | All sites | AT70 |
| *Guizotia abyssinica* (L. f.) Cass. [Asteraceae] | Nug (A) | H | Se | Leech (Alqit) | Infusion | Oral | C, MA | AT91 |
| *Laggera crispata* (Vahl) Hepper & Wood. [Asteraceae] | Ge-fon-foo (S) | H | L | Wound, Swelling | Crushed | Topical | C, ME | AT112 |
| *Senecio hadiensis* Forssk. [Asteraceae] |  | C | L | Eye infection (Wucher) | Extract the juice | Drops into the eyes | C | AT220 |
| *Tanacetum cinerariifolium* (Trev.) Sch. Bip. [Asteraceae] | Pyrethrine (A) | H | Fl (Young) | Ectoparasites | Crushed | Wash | MA | AT221 |
| *Vernonia amygdalina* Del. [Asteraceae] | Gola (G), Heba (S, M) | S | L, St | Diarrhea, Increase milk production, Fattening (Calf) | Crushed | Oral | All sites | AT206 |
| *Vernonia myriantha* Hook. f. [Asteraceae] | Dengrita, Aguaje(G, Q) | S | L | Ectoparasites | Crushed | Wash | ME, Q | AT205 |
|  |  |  | L, B | Leech (Alqit), Sudden illness (Dengetegan), | Infusion | Oral, Nasal |  |  |
| *Vernonia subligera* O. Hoffm. [Asteraceae] | Ereja (G) | S | L | Leech (Alqit) | Extract the juice | Nasal | Q, W, MA, SI | AT207 |
| *Vernonia* sp. [Asteraceae] | Merar (A) | H | L | Eye infection (Wucher) | Extract the juice | Drops into the eyes | ME | AT222 |
|  |  |  |  | Flu | Crushed | Oral |  |  |
| *Balanites aegyptiaca* (L.) Del. [Balanitaceae] | Bedeno (A, G) | T | L | Eye infection | Extract the juice | Drops into the eyes | MQ | AT22 |
| *Impatiens tinctoria* A. Rich. [Balsaminaceae] | Inshoshela (A) | H | L | Eye infection | Infusion | Drops into the eyes | Q, MA | AT101 |
| *Carica papaya* L. [Caricaceae] | Papaya (A) | T | L | Bloat, Anthra (Sheme etere) (Yewerche-bashe), Retained placenta | Infusion | Oral, Topical | MA | AT34 |
| *Kalanchoe densiflora* Rolfe [Crassulaceae] | Andahula (A) | H | L | Swelling | Crushed | Topical | ME, MA, SO | AT107 |
| *Brassica carinata* A. Br. [Brassicaceae] | Gomen zere (A) | H | Se | Urine retention | Infusion | Oral | W | AT27 |
|  |  |  |  | Bloat | Infusion | Oral |  |  |
| *Terminalia schimperiana* Hochst. [Combretaceae] | Seba (Q) | T | L | Bloat, Internal parasite | Infusion | Oral | Q | AT223 |
| *Cucumis ficifolius* A. Rich. [Cucurbitaceae] | Hulgerecho (M), Adene debaqula (Q), Yemeder qimbiba, Yafer-granger (G), Yale-tay (Si) | H | R | Bloat, Anthrax (Shem-itere), Trypanosomosis | Infusion | Oral | All sites | AT54 |
| *Lagenaria abyssinica* (Hook. f.) C. Jeffrey [Cucurbitaceae] |  | H | L | Evil spirit | Crushed, Cut small part | Topical,  tied | MA | AT224 |
| *Momordica foetida* Schumach. [Cucurbitaceae] | Araret, Tere (G, S), Yehonzet beye (G) | H | L | Diarrhea, Evil sprit | Infusion | Oral | ME, MA, SI, SO | AT130 |
|  |  |  | R | Snake bite | Infusion | Oral |  |  |
| *Euclea divinorum* Hiern. [Ebenaceae] | Migiyar, Mesa,Meqnesa (G) | T | B | Eye infection | Infusion, Crushed | Drops into the eyes, topical | C, SO | AT77 |
| *Clutia abyssinica* Kaub. & Spach. [Euphorbiaceae] | Yemar semat (G) | S | L | Internal parasite | Infusion | Oral | SI, SO | AT46 |
| *Croton macrostachyus* Del. [Euphorbiaceae] | Mekenisa (G) | T | L, B | Sudden illness (Dengetegan), Indigestion (due to swallowing plastic material), Blackleg | Infusion | Oral | All sites | AT53 |
| *Ricinus communis* L. [Euphorbiaceae] | Gulo (G) | S | Se | Internal parasite | Infusion | Oral | W, MQ, MA | AT169 |
| *Tragia cinerea* (Pax) M.G.Gilbert & Radcl.-Smith [Euphorbiaceae] | Dobiye, Neger (G, Q) | H | L | Anthrax (Shem-itere (G)) | Infusion | Oral | Q, SO, MA | AT225 |
| *Acacia seyal* Del. [Fabaceae] | Wacho-gerar (G) | T | B | Bloat | Infusion | Oral | W, MA | AT03 |
|  |  |  | B | Eye infection | Extract the juice | Drops into the eyes |  |  |
| *Calpurnia aurea* (Ait.) Benth. [Fabaceae] | Zegnet, Singo (G), Ticho (S) | S | L | Ectoparasites | Crushed | Wash | C, Q, W, MQ, MA, SI, SO | AT31 |
| *Crotalaria incana* L. [Fabaceae] |  | H | L | Sudden illness (Dengetegan) | Infusion | Oral | ME | AT52 |
| *Erythrina brucei* Schweinf. [Fabaceae] | Burat, Weleya (Q, G) | T | B | Ectoparasites | Crushed | Wash | C, ME | AT226 |
|  |  |  | B | Diarrhea | Infusion | Oral |  |  |
| *Millettia ferruginea* (Hochst.) Bak. [Fabaceae] | Berebera (A) | T | L | Ectoparasites | Crushed | Wash | W | AT227 |
| *Senna septemtrionalis* (Viv.) Irwin & Bameby [Fabaceae] | Sememeki (G, S) | S | L | Sudden illness (Dengetegan), Snake bite | Infusion | Oral | ME, MA, SI, SO | AT183 |
| *Hypericum quartinianum* A. Rich. [Hypericaceae] | Ansereche (G) | S | L | Diarrhea | Infusion | Oral | C | AT228 |
| *Fuerstia africana* T.C.E. Fr. [Lamiaceae] | Yegiye ensosla (G), Nazoli (S), Hureda (M) | H | L | Eye infection/ injury (Wucher) | Extract the juice | Drops into the eyes | Q, W | AT86 |
| *Leonotis* *ocymifolia* (Burm. f.) Iwarsson [Lamiaceae] | Chenbolibi (S) | S | L | Diarrhea, Internal parasites | Infusion | Oral | MA, SI | AT116 |
| *Leucas* *argentea* Gurke [Lamiaceae] | Fiza, Kiza (G) | H | L | Internal parasites | Infusion | Oral | C, W, ME, MA, SI | AT118 |
| *Leucas* *martinicensis* (Jacq.) R. Br. [Lamiaceae] | Mangorbecha (G) | H | L | Bloat | Infusion | Oral | ME, MA | AT229 |
| *Ocimum urticifolium* Roth [Lamiaceae] | Yelebe fuanfa, Delibekera (Q) | S | L | Retained placenta | Infusion | Oral | W, MQ, ME, SI | AT137 |
| *Plectranthus* *cylindraceus* Hochst. ex Benth. [Lamiaceae] | Qintele sat (G) | H | L | Evil sprit | Crushed | Topical | C, W, ME | AT152 |
| *Salvia* *tiliifolia* Vahl [Lamiaceae] | Hurdu (Q) | H | L | Eye infection | Infusion | Drops into the eyes | Q | AT230 |
| *Satureja* *abyssinica* (Benth.) Briq. [Lamiaceae] | Tebeqo (G) | H | L, R | Internal parasite, bloat | Infusion | Oral | MQ, ME, MA, SI, SO | AT178 |
| *Commelina* *latifolia* Hochst. et A. Rich. [Commelinaceae] | Gemete, Laloncha (G) | H | L | Retained placenta, increase milk production | Crushed | Oral (Eat) | Q, ME, | AT231 |
| *Tapinanthus* *globiferus* (A. Rich.) Tieghem [Loranthaceae] | Teqetla (A) (growing on coffee, chat, peach) | S | Whole plant | Anthrax (Shem-itere (G)), Blackleg (Yewerche bashe (G)), Sudden illness (Dengetegan) | Crushed | Wash | ME, MA, SI, SO | AT198 |
| *Sida* *rhombifolia* L. [Malvaceae] | Badefacha (S, M) | H | R | Fattening, Diarrhea (Calf) | Infusion | Oral | C | AT184 |
| *Sida* *schimperiana* Hochst. ex A. Rich. [Malvaceae] | Chifereg (A), Anjajewet (G) | S | Whole part | Indigestion, Bloat | Infusion | Oral | MQ, ME, SO | AT185 |
| *Ekebergia capensis* Sparrm. [Meliaceae] | Wulel (S), Guareba (G) | T | L | Animal bite by hyena (horse, donkey, cattle) | Crushed | Topical | C, ME, MA | AT71 |
|  |  |  | L | Bloat | Infusion | Oral |  |  |
| *Bersama* *abyssinica* Fresen. [Melianthaceae] | Hureta (G) | T | L | Ectoparasites | Crushed | Wash | MA | AT24 |
| *Stephania* *abyssinica* (Dillon & A.Rich.) Walp. [Menispermaceae] | Foreformat , Kelalla (G), Meqeres (S) | C | R | Anthrax (Shem-itere (G)), Bad evil, Sudden illness (Dengetegan), | Infusion | Oral | C, MQ, ME, MA, SI, SO | AT195 |
|  |  |  | R, L | African horse sickness | Infusion | Oral |  |  |
| *Ficus sycomorus* L. [Moraceae] | Wedisha (G) | T | L | Bloat | Infusion | Oral | C, M | AT83 |
| *Ensete ventricosum* (Welw.) Cheesman [Musaceae] | Eset (G, S) | S | C, L | Retained placenta | Crushed | oral | C, Q, W, ME, MA, SI, SO |  |
| *Eucalyptus globulus* Labill. [Myrtaceae] | Antakirt (G) | T | L (Young) | Anthrax (Shem-itere (G)) | Infusion | Oral | C, Q, ME, MA, SI, SO | AT76 |
| *Argemone mexicana* L. [Papaveraceae] | Nech-lebash (A) | H | L | Indigestion (Calf) | Decoction | Oral | MQ, SI | AT17 |
| *Phytolacca dodecandra* L’Herit. [Phytolaccaceae] | Endod (A) | S | L (Young) | Leech (Alqit) | Extract the juice | Nasal | C, W, ME, SO | AT148 |
|  |  |  | L | Cough | Infusion | Oral |  |  |
|  |  |  | R | Vaccine for Rabies (cattles) | Infusion | Oral |  |  |
| *Pittosporum viridiflorum* Sis [Pittosporaceae] | Hunbosho (Si), Ulaga (G) | T | L | Anthrax (Shem-itere (G)), Blackleg (Yewerche bashe (G)) | Infusion | Oral |  | AT149 |
| *Persicaria senegalensis* (Meisn.) Sojak [Polygonaceae] | Hobet (S), Nech azhe (G) | H | L | Retained placenta | Infusion/decoction | Oral | C, W, MQ, SI | AT146 |
| *Cymbopogon citratus* (DC. ex Nees) Stapf [Poaceae] | Hiticho (M), Deg sar (G, S), Moseret (G)) | H | L | Sudden illness (Dengetegan); Anthrax | Infusion | Oral | MQ, MA, SO | AT57 |
| *Prunus persica* (L.) Batsch [Rosaceae] | Kuk (A) | T | L | Sudden illness (Dengetegan) | Infusion | Oral | C, MA | AT158 |
| *Rosa abyssinica* Lindley [Rosaceae] | Engorcha(G) | S | Fl | Eye infection | Extract the juice | Drops into the eye | ME | AT232 |
| *Pentas schimperiana* (A. Rich.) Vatke [Rubiaceae] | Mesa-bur (Q) | S | L, R | Bone fracture (Calf) | Infusion/decoction | Oral | C | AT144 |
| *Citrus aurantifolia* (Christm.) Swingle [Rutaceae] | Lomi (A) | T | Fr | Leech (Alqit), Sudden illness (Dengetegan) | Extract the juice | Nasal | C, Sl | AT39 |
|  |  |  |  | Emergency | Infusion | Oral |  |  |
| *Citrus aurantium* L. [Rutaceae] | Hometate (A) | T | Fr | Leech (Alqit), Sudden illness (Dengetegan) | Extract the juice | Oral, nasal | Sl | AT40 |
| *Clausena anisata* (Willd.) Benth. [Rutaceae] | Lemuche (A) | T | L | Sudden illness (Dengetegan) | Infusion | Oral | MA | AT42 |
| *Dobera glabra* (Forssk.) poir. [Salvadoraceae] | Alinquba(M) | S | L | Ectoparasites | Crushed | wash | MQ | AT233 |
| *Osyris quadripartita* Decn. [Santalaceae] | Mekeker (G); Qeret(A) | T | L | Bloat, Diarrhea | Infusion | Oral | C, Q | AT234 |
| *Dodonaea angustifolia* L. f. [Sapindaceae] | Keteketa (A) | S | L | Bloat(Calf), Fattening (Calf) | Infusion | Oral | C, Q, W, MQ, ME, SO | AT235 |
|  |  |  | L | Ectoparasites | Crushed | Wash |  |  |
| *Verbascum sinaiticum* Benth. [Scrophulariaceae] | Yemar enzir (G), Halemecha, Huleten huta (M), Yumar amel ( Q, S) | H | R, L | Urinary retention, Indigestion (calf), Sudden illness (Dengetegan), | Infusion | Oral | C, W, MQ, ME, MA, SI, SO | AT203 |
| *Datura inoxia* Mill. [Solanaceae] |  | H | L | Bloat | Infusion | Oral | ME | AT236 |
| *Datura stramonium* L. [Solanaceae] | Mechara (M, S), Azaza (G, Q) | H | L | Bloat | Infusion | Oral | C, Q, W, MQ, ME, MA, SI, SO | AT63 |
|  |  |  | Se | Wound (Horse, Cattle) | Crushed | Topical |  |  |
| *Discopodium penninervium* Hochst. [Solanaceae] | Enchochika (G) | T | L | Bloat | Infusion | Oral | C, ME, SO | AT65 |
| *Nicotiana tabacum* L. [Solanaceae] | Tenbaho (A) | H | L | Leech (Alqit) | Infusion | Nasal | All sites | AT237 |
|  |  |  |  | Ectoparasites | Crushed | Wash |  |  |
|  |  |  |  | Bloat, Cough (Sheep), Fattening | Infusion | Oral |  |  |
| *Solanecio gigas* (Vatke) C. Jeffrey [Solanaceae] | Tonbi (G), Yezogare gaje (Q) | S | L | Sudden illness (Dengetegan) | Infusion | Oral | Q, MA | AT189 |
|  |  |  | R | Internal parasite | Infusion | Oral |  |  |
| *Solanecio mannii* (Hook. f.) C. Jeffrey [Solanaceae] | Gemar (G) | S | L | Anthrax (Shem-itere (G)), Blackleg (Yewerche bashe (G)) | Infusion | Oral | C, MA, SO | AT190 |
| *Solanum incanum* L. [Solanaceae] | Embuay (A), Zereche (G), Yaqom-zaro (S) | S | L, R | Anthrax (Shem-itere (G)), Retained placenta, Sudden illness (Dengetegan) | Infusion | Oral | All sites | AT193 |
|  |  |  | Fr | Wound, Ectoparasites | Crushed | Topical |  |  |
| *Solanum marginatum* L.f. [Solanaceae] | Yewelel engorcha (S) | S | L | Sudden illness (Dengetegan) | Crushed | Wash | SI | AT238 |
| *Solanum nigrum* L. [Solanaceae] | Emberebuniye (G) | H | R,L | Sudden illness (Dengetegan) | Infusion | Oral | C | AT194 |
| *Tacca leontopetaloides* (L.) O. Ktze. [Taccaceae] | Kureshe (G) | H | Tu | Anthrax (Shem-itere (G)) | Infusion | Oral | C, MA | AT239 |
| *Gnidia* *stenophylla* Gilg [Thymelaceae] | Mesemes (G) | H | R | Retained placenta | Infusion | Oral | C, SO | AT90 |
| *Grewia ferruginea* Hochst. ex A. Rich. [Tiliaceae] |  | S | L | Retained placenta | Infusion | Oral | MA | AT240 |
| *Urera hypselodendron* (A. Rich.) Wedd. [Urticaceae] | Alila, Abila (Q) | C | L | Indigestion | Infusion | Oral | MA | AT241 |
| *Ampelocissus bombycina* (Bak.)Planch. [Vitaceae] |  | H | R | Bloat, Black leg, Anthrax (Shem-itere), | Infusion | Oral | Q | AT242 |
| *Cyphostemma adenanthum* (Fresen.) descoings [Vitaceae] | Gidila( M) | H | R, L | Anthrax (Shem-itere (G)), Blackleg (Yewerche bashe (G)) | Infusion | Oral | MQ | AT243 |
| *Cyphostemma cyphopetalum* (Fresen.) Desc. Ex Wild & R.B. Dr umm [Vitaceae] | Toleje (G) | C | L | Wound | Crushed | Topical | ME, MA, SI | AT59 |
| *Cyphostemma niveum* (Hochst. Ex Schweinf.) Desc.[Vitaceae] | Yeseb eje, Lequmye, Gerechet (G) | C | L | Wound, Swelling | Crushed | Topical | ME, MA, SI | AT60 |
