# Supplementary material for: Medicinal plant use practice in four ethnic communities (Gurage, Mareqo, Qebena, and Silti), south central Ethiopia
Source: J Ethnobiol Ethnomed. 2020 May 24;16:27. doi: 10.1186/s13002-020-00377-1 (PMC7245860; doi:10.1186/s13002-020-00377-1)
Supplement: Supplementary file 3 — Additional file 3. Rank order priority (ROP) values of culturally important species (ROP ≥50%) major uses, relative popularity level and study sites [file 13002_2020_377_MOESM3_ESM.docx]

**Additional file 3.** Rank order priority (ROP) values of culturally important species (ROP ≥50%) major uses, relative popularity level and study sites.

| **Important plants** | **Major uses** | **Iu** | **Ip** | **FL** | **RPL** | **ROP** | **Study site** |
| --- | --- | --- | --- | --- | --- | --- | --- |
| *Ajuga integrifolia* | abdominal pain | 24.0 | 24.0 | 100.0 | 1.0 | 100.0 | Sodo |
| *Apodytes dimidiate* | abdominal pain, diarrhea, cholera | 8.0 | 8.0 | 100.0 | 0.7 | 66.7 | Sodo |
|  | abdominal pain, diarrhea, cholera | 18.0 | 17.0 | 94.4 | 1.0 | 94.4 | Meskan |
| *Artemisia abyssinica* | abdominal pain, diarrhea | 22.0 | 17.0 | 77.3 | 1.0 | 77.3 | Meskan |
| *Clerodendrum myricoides* | abdominal pain, diarrhea | 20.0 | 19.0 | 95.0 | 1.0 | 95.0 | Cheha |
|  | abdominal pain, diarrhea | 25.0 | 25.0 | 100.0 | 1.0 | 100.0 | Qebena |
| *Cucumis ficifolius* | abdominal pain, diarrhea | 11.0 | 6.0 | 54.5 | 0.9 | 50.0 | Sodo |
| *Cymbopogon citratus* | abdominal pain, diarrhea | 12.0 | 8.0 | 66.7 | 0.9 | 57.1 | Mareqo |
| *Echinops kebericho* | abdominal pain, diarrhea | 16.0 | 10.0 | 62.5 | 1.0 | 62.5 | Silti |
|  | abdominal pain/bloating | 18.0 | 14.0 | 77.8 | 1.0 | 77.8 | Mareqo |
|  | amoebiasis, abdominal pain | 28.0 | 24.0 | 85.7 | 1.0 | 85.7 | Qebena |
| *Hagenia abyssinica* | intestinal parasite, taeniasis | 6.0 | 6.0 | 100.0 | 0.9 | 85.7 | Wulbareg |
|  | intestinal parasite, taeniasis | 13.0 | 13.0 | 100.0 | 1.0 | 100.0 | Sodo |
|  | intestinal parasite, taeniasis | 9.0 | 7.0 | 77.8 | 0.7 | 53.8 | Cheha |
|  | intestinal parasite, taeniasis | 19.0 | 18.0 | 94.7 | 1.0 | 94.7 | Meskan |
| *Lepidium sativum* | abdominal pain | 12.0 | 9.0 | 75.0 | 0.8 | 60.0 | Meskan |
|  | abdominal pain | 16.0 | 8.0 | 50.0 | 1.0 | 50.0 | Silti |
|  | abdominal pain | 10.0 | 5.0 | 50.0 | 1.0 | 50.0 | Wulbareg |
| *Lippia adoensis* var *adoensis* | diarrhea, abdominal pain | 5.0 | 5.0 | 100.0 | 0.7 | 71.4 | Wulbareg |
|  | diarrhea, abdominal pain | 10.0 | 9.0 | 90.0 | 0.7 | 60.0 | Silti |
|  | diarrhea, abdominal pain | 17.0 | 11.0 | 64.7 | 1.0 | 64.7 | Mareqo |
| *Ocimum lamiifolium* | amoebiasis, abdominal pain | 27.0 | 17.0 | 63.0 | 1.0 | 63.0 | Silti |
| *Rumex nepalensis* | abdominal pain | 18.0 | 16.0 | 88.9 | 1.0 | 88.9 | Meskan |
| *Ruta chalepensis* | abdominal pain | 20.0 | 13.0 | 65.0 | 1.0 | 65.0 | Silti |
|  | abdominal pain | 9.0 | 9.0 | 100.0 | 1.0 | 100.0 | Wulbareg |
|  | diarrhea | 22.0 | 13.0 | 59.1 | 1.0 | 59.1 | Mareqo |
|  | diarrhea, abdominal pain | 27.0 | 17.0 | 63.0 | 1.0 | 63.0 | Qebena |
|  | abdominal blotting | 9.0 | 5.0 | 55.6 | 1.0 | 55.6 | Wulbareg |
| *Solanum incanum* | abdominal pain | 13.0 | 13.0 | 100.0 | 0.9 | 86.7 | Silti |
|  | abdominal pain | 11.0 | 11.0 | 100.0 | 1.0 | 100.0 | Wulbareg |
|  | abdominal pain | 17.0 | 11.0 | 64.7 | 1.0 | 64.7 | Meskan |
|  | abdominal pain | 10.0 | 7.0 | 70.0 | 0.7 | 50.0 | Mareqo |
| *Verbascum sinaiticum* | amoebiasis | 19.0 | 12.0 | 63.2 | 1.0 | 63.2 | Silti |
|  | abdominal pain | 13.0 | 11.0 | 84.6 | 0.9 | 73.3 | Meskan |
|  | diarrhea | 29.0 | 23.0 | 79.3 | 1.0 | 79.3 | Mareqo |
| *Verbena officinalis* | abdominal pain, diarrhea | 11.0 | 11.0 | 100.0 | 0.8 | 78.6 | Muhir-Aklil |
| *Vernonia amygdalina* | ascariasis | 17.0 | 9.0 | 52.9 | 1.0 | 52.9 | Silti |
|  | abdominal pain, intestinal parasites | 14.0 | 7.0 | 50.0 | 1.0 | 50.0 | Sodo |
| *Acacia seyal* | indigestion | 13.0 | 13.0 | 100.0 | 1.0 | 100.0 | Wulbareg |
| *Bridelia micrantha* | indigestion | 15.0 | 15.0 | 100.0 | 1.0 | 100.0 | Qebena |
| *Echinops kebericho* | indigestion | 12.0 | 8.0 | 66.7 | 0.8 | 53.3 | Meskan |
| *Ficus vasta* | indigestion | 18.0 | 18.0 | 100.0 | 1.0 | 100.0 | Qebena |
| *Leucas argentea* | indigestion | 27.0 | 17.0 | 63.0 | 1.0 | 63.0 | Silti |
|  | indigestion | 22.0 | 17.0 | 77.3 | 1.0 | 77.3 | Meskan |
|  | indigestion | 17.0 | 16.0 | 94.1 | 1.0 | 94.1 | Muhir-Aklil |
|  | indigestion | 11.0 | 11.0 | 100.0 | 0.8 | 84.6 | Cheha |
| *Maytenus heterophylla* | indigestion | 15.0 | 15.0 | 100.0 | 1.0 | 100.0 | Qebena |
| *Myrica salicifolia* | indigestion | 21.0 | 21.0 | 100.0 | 1.0 | 100.0 | Muhir-Aklil |
| *Satureja abyssinica* | indigestion | 16.0 | 11.0 | 68.8 | 1.0 | 68.8 | Meskan |
|  | indigestion | 7.0 | 7.0 | 100.0 | 0.6 | 58.3 | Sodo |
| *Stephania abyssinica* | indigestion | 3.0 | 2.0 | 66.7 | 0.9 | 84.0 | Cheha |
| *Verbena officinalis* | indigestion | 21.0 | 21.0 | 100.0 | 1.0 | 100.0 | Meskan |
|  | indigestion | 28.0 | 24.0 | 85.7 | 1.0 | 85.7 | Silti |
|  | indigestion | 21.0 | 15.0 | 71.4 | 1.0 | 71.4 | Cheha |
|  | indigestion | 24.0 | 13.0 | 54.2 | 1.0 | 54.2 | Mareqo |
| *Zea mays* | indigestion | 9.0 | 9.0 | 100.0 | 0.6 | 64.3 | Mareqo |
| *Acacia abyssinica* | tonsilitis | 11.0 | 9.0 | 81.8 | 1.0 | 81.8 | Wulbareg |
| *Acmella caulirhiza* | tonsilitis | 13.0 | 11.0 | 84.6 | 0.9 | 73.3 | Meskan |
|  | tonsilitis | 11.0 | 9.0 | 81.8 | 0.8 | 64.3 | Muhir-Aklil |
| *Allium sativum* | common cold | 18.0 | 10.0 | 55.6 | 1.0 | 55.6 | Cheha |
|  | common cold | 8.0 | 5.0 | 62.5 | 1.0 | 62.5 | Wulbareg |
| *Artemisia abyssinica* | pneumonia, strong cough | 20.0 | 19.0 | 95.0 | 1.0 | 95.0 | Silti |
| *Catha edulis* | tonsilits | 11.0 | 10.0 | 90.9 | 0.8 | 76.9 | Cheha |
|  | tonsillitis | 20.0 | 20.0 | 100.0 | 1.0 | 100.0 | Qebena |
| *Ocimum lamiifolium* | common cold | 27.0 | 17.0 | 63.0 | 1.0 | 63.0 | Meskan |
|  | common cold | 23.0 | 23.0 | 100.0 | 1.0 | 100.0 | Muhir-Aklil |
|  | common cold | 21.0 | 19.0 | 90.5 | 1.0 | 90.5 | Cheha |
|  | common cold | 13.0 | 9.0 | 69.2 | 1.0 | 69.2 | Wulbareg |
|  | common cold | 22.0 | 22.0 | 100.0 | 1.0 | 100.0 | Qebena |
|  | common cold | 29.0 | 28.0 | 96.6 | 1.0 | 96.6 | Mareqo |
|  | pneumonia | 27.0 | 24.0 | 88.9 | 1.0 | 88.9 | Silti |
| *Pittosporum viridiflorum* | pneumonia, TB, strong cough | 13.0 | 13.0 | 100.0 | 1.0 | 100.0 | Wulbareg |
| *Plantago lanceolata* | tonsillitis | 9.0 | 9.0 | 100.0 | 0.6 | 64.3 | Mareqo |
| *Rumex nepalensis* | tonsillitis | 8.0 | 5.0 | 62.5 | 1.0 | 62.5 | Wulbareg |
| *Ruta chalepensis* | common cold | 20.0 | 11.0 | 55.0 | 1.0 | 55.0 | Silti |
| *Foeniculum vulgare* | gonorrhea, urinary retention | 9.0 | 9.0 | 100.0 | 0.6 | 60.0 | Qebena |
|  | gonorrhea, urinary retention | 19.0 | 17.0 | 89.5 | 1.0 | 89.5 | Mareqo |
|  | gonorrhea, urinary retention | 4.0 | 4.0 | 100.0 | 0.6 | 57.1 | Wulbareg |
|  | gonorrhea, urinary retention | 14.0 | 7.0 | 50.0 | 1.0 | 50.0 | Cheha |
|  | gonorrhea, urinary retention | 21.0 | 20.0 | 95.2 | 1.0 | 95.2 | Silti |
| *Lepidium sativum* | gonorrhea, urinary retention | 16.0 | 8.0 | 50.0 | 1.0 | 50.0 | Cheha |
|  | gonorrhea, urinary retention | 16.0 | 14.0 | 87.5 | 1.0 | 87.5 | Qebena |
| *Rumex abyssinicus* | urinary retention, gonorrhea | 22.0 | 14.0 | 63.6 | 1.0 | 63.6 | Silti |
| *Aloe pubescens* | rehumatism, stabbing pain | 10.0 | 7.0 | 70.0 | 0.7 | 50.0 | Muhir-Aklil |
| *Ajuga integrifolia* | rehumatism, stabbing pain | 27.0 | 27.0 | 100.0 | 1.0 | 100.0 | Meskan |
|  | rehumatism, stabbing pain | 19.0 | 16.0 | 84.2 | 1.0 | 84.2 | Mareqo |
|  | rheumatism | 25.0 | 20.0 | 80.0 | 1.0 | 80.0 | Cheha |
| *Ruta chalepensis* | rheumatism | 21.0 | 17.0 | 81.0 | 1.0 | 81.0 | Cheha |
| *Aloe pubescens* | wound | 12.0 | 6.0 | 50.0 | 1.0 | 50.0 | Wulbareg |
| *Argemone Mexicana* | dandruff | 18.0 | 18.0 | 100.0 | 1.0 | 100.0 | Silti |
| *Clematis simensis* | eczema | 11.0 | 9.0 | 81.8 | 0.9 | 75.0 | Sodo |
| *Croton macrostachyus* | wound | 14.0 | 8.0 | 57.1 | 1.0 | 57.1 | Wulbareg |
|  | wound, blood clotting | 30.0 | 21.0 | 70.0 | 1.0 | 70.0 | Silti |
|  | blood clotting | 19.0 | 15.0 | 78.9 | 1.0 | 78.9 | Cheha |
| *Datura stramonium* | dandruff | 10.0 | 7.0 | 70.0 | 0.8 | 58.3 | Sodo |
| *Kalanchoe densiflora* | wound | 9.0 | 7.0 | 77.8 | 0.6 | 50.0 | Muhir-Aklil |
| *Lagenaria siceraria* | dandruff | 8.0 | 8.0 | 100.0 | 0.6 | 61.5 | Cheha |
|  | dandruff | 17.0 | 15.0 | 88.2 | 1.0 | 88.2 | Qebena |
| *Plantago lanceolata* | wound | 8.0 | 8.0 | 100.0 | 1.0 | 100.0 | Wulbareg |
| *Rumex nepalensis* | wound | 13.0 | 9.0 | 69.2 | 1.0 | 69.2 | Cheha |
| *Salvia nilotica* | wound | 16.0 | 16.0 | 100.0 | 1.0 | 100.0 | Meskan |
|  | wound | 8.0 | 6.0 | 75.0 | 0.7 | 50.0 | Sodo |
| *Xanthium strumarium* | tinea versicolor | 10.0 | 9.0 | 90.0 | 0.7 | 64.3 | Mareqo |
| *Pycnostachys abyssinica* | eye infection | 16 | 8 | 50.0 | 1.0 | 50.0 | Silti |
| *Lens culinaris* | herpes zoster | 10 | 10 | 100.0 | 0.7 | 66.7 | Meskan |
| *Phytolacca dodecandra* | rabies | 15 | 11 | 73.3 | 1.0 | 73.3 | Sodo |
| *Artemisia afra* | headache | 20.0 | 13.0 | 65.0 | 1.0 | 65.0 | Cheha |
|  | headache | 30.0 | 30.0 | 100.0 | 1.0 | 100.0 | Qebena |
|  | headache | 17.0 | 9.0 | 52.9 | 1.0 | 52.9 | Muhir-Aklil |
| *Allium sativum* | malaria | 30.0 | 20.0 | 66.7 | 1.0 | 66.7 | Qebena |
| *Carica papaya* | malaria | 15.0 | 15.0 | 100.0 | 1.0 | 100.0 | Silti |
|  | malaria | 12.0 | 12.0 | 100.0 | 0.9 | 85.7 | Mareqo |
|  | malaria | 24.0 | 24.0 | 100.0 | 1.0 | 100.0 | Qebena |
| *Justicia schimperiana* | malaria | 23.0 | 12.0 | 52.2 | 1.0 | 52.2 | Meskan |
| *Nigella sativa* | headache | 16.0 | 15.0 | 93.8 | 1.0 | 93.8 | Qebena |
| *Pycnostachys abyssinica* | malaria | 7.0 | 4.0 | 57.1 | 1.0 | 57.1 | Wulbareg |
| *Vernonia amygdalina* | malaria | 18.0 | 9.0 | 50.0 | 1.0 | 50.0 | Mareqo |
| *Acmella caulirhiza* | toothache | 18 | 12 | 66.7 | 1.0 | 66.7 | Cheha |
| *Allium sativum* | toothache | 18 | 10 | 55.6 | 1.0 | 55.6 | Cheha |
| *Ekebergia capensis* | toothache | 7 | 4 | 100.0 | 0.9 | 100.0 | Meskan |
| *Datura stramonium* | toothache | 10 | 10 | 100.0 | 1.0 | 100.0 | Wulbareg |
|  | toothache | 9 | 9 | 100.00 | 0.60 | 60 | Qebena |
| *Gladiolous abyssinicus* | toothache | 23.0 | 18.0 | 78.3 | 1.0 | 78.3 | Cheha |
| *Ocimum urticifolium* | toothache | 16 | 14 | 87.5 | 1.0 | 87.5 | Silti |
| *Olea europaea* subsp. *cuspidate* | toothache | 6 | 6 | 100.0 | 0.5 | 50.0 | Sodo |
| *Olinia rochetiana* | toothache | 8 | 8 | 100.0 | 0.6 | 61.5 | Cheha |
|  | toothache | 8 | 8 | 100.00 | 0.53 | 53 | Qebena |
| *Premna schimperi* | toothache | 8 | 8 | 100.0 | 0.5 | 53.3 | Silti |
| *Hypoestes forskaolii* | retained placenta | 18.0 | 12.0 | 66.7 | 1.0 | 66.7 | Silti |
| *Juniperus procera* | after birth pains | 12.0 | 12.0 | 100.0 | 0.8 | 80.0 | Silti |
|  | retained placenta | 9.0 | 9.0 | 100.0 | 0.6 | 64.3 | Mareqo |
| *Linum ustitatissimum* | retained placenta | 13.0 | 13.0 | 100.0 | 0.9 | 92.9 | Mareqo |
| *Plectranthus minutiflorus* | after birth pains | 11.0 | 11.0 | 100.0 | 1.0 | 100.0 | Wulbareg |
| *Acacia seyal* | liver complaints (jaundice) | 9.0 | 9.0 | 100.0 | 0.8 | 75.0 | Sodo |
| *Cucumis ficifolius* | liver complaints (jaundice) | 16.0 | 12.0 | 75.0 | 1.0 | 75.0 | Mareqo |
| *Ensete ventricosum* | liver complaints (jaundice) | 17.0 | 14.0 | 66.7 | 1.0 | 84.0 | Cheha |
|  | liver complaints (jaundice) | 9.0 | 9.0 | 100.0 | 0.6 | 60.0 | Qebena |
| *Justicia schimperiana* | liver complaints (jaundice) | 16.0 | 13.0 | 81.3 | 1.0 | 81.3 | Sodo |
|  | liver complaints (jaundice) | 14.0 | 14.0 | 100.0 | 1.0 | 100.0 | Muhir-Aklil |
| *Calvatia* sp. [Agaricaceae] | liver complaints (jaundice) | 23.0 | 23.0 | 100.0 | 1.0 | 100.0 | Silti |
|  | liver complaints (jaundice) | 9.0 | 9.0 | 100.0 | 1.0 | 100.0 | Wulbareg |
| *Brassica carinata* | anthrax | 13.0 | 11.0 | 84.6 | 0.9 | 78.6 | Muhir-Aklil |
| *Brassica nigra* | anthrax | 17.0 | 17.0 | 100.0 | 1.0 | 100.0 | Qebena |
| *Colocasia esculenta* | anthrax | 11.0 | 9.0 | 81.8 | 0.8 | 64.3 | Muhir-Aklil |
| *Cucumis ficifolius* | anthrax | 17.0 | 9.0 | 52.9 | 1.0 | 52.9 | Cheha |
|  | anthrax | 11.0 | 8.0 | 72.7 | 0.7 | 53.3 | Qebena |
| *Cymbopogon citratus* | anthrax | 22.0 | 22.0 | 100.0 | 1.0 | 100.0 | Qebena |
| *Polygala sadebeckiana* | anthrax | 24.0 | 21.0 | 66.7 | 1.0 | 84.0 | Cheha |
|  | anthrax | 17.0 | 12.0 | 70.6 | 1.0 | 70.6 | Muhir-Aklil |
|  | anthrax | 27.0 | 20.0 | 74.1 | 1.0 | 74.1 | Qebena |
| *Ajuga integrifolia* | anorexia (loss of appetite) | 25.0 | 21.0 | 84.0 | 1.0 | 84.0 | Cheha |
|  | anorexia (loss of appetite) | 28.0 | 23.0 | 82.1 | 1.0 | 82.1 | Qebena |
|  | anorexia (loss of appetite) | 26.0 | 19.0 | 73.1 | 1.0 | 73.1 | Muhir-Aklil |
| *Agave sisaliana* | general malaise (michi) | 12.0 | 5.0 | 100.0 | 0.9 | 100.0 | Meskan |
| *Allium sativum* | general malaise (michi) | 27.0 | 14.0 | 51.9 | 1.0 | 51.9 | Muhir-Aklil |
|  | general malaise (michi) | 18.0 | 12.0 | 66.7 | 1.0 | 66.7 | Silti |
|  | general malaise (michi) | 25.0 | 14.0 | 56.0 | 1.0 | 56.0 | Mareqo |
|  | general malaise (michi) | 8.0 | 5.0 | 62.5 | 1.0 | 62.5 | Wulbareg |
|  | general malaise (michi) | 18.0 | 10.0 | 55.6 | 1.0 | 55.6 | Cheha |
|  | general malaise (michi) | 18.0 | 10.0 | 55.6 | 1.0 | 55.6 | Cheha |
| *Aloe pubescens* | general malaise (michi) | 10.0 | 9.0 | 90.0 | 0.7 | 64.3 | Muhir-Aklil |
|  | general health (boost infant’s immunity) | 15.0 | 12.0 | 80.0 | 1.0 | 80.0 | Meskan |
|  | general health (boost infant’s immunity) | 29.0 | 26.0 | 89.7 | 1.0 | 89.7 | Qebena |
|  | localized swelling | 10.0 | 7.0 | 70.0 | 0.7 | 50.0 | Muhir-Aklil |
| *Artemisia abyssinica* | evil sprit | 15.0 | 11.0 | 73.3 | 1.0 | 73.3 | Sodo |
| *Clerodendrum myricoides* | evil spirit | 27.0 | 10.0 | 51.9 | 1.0 | 51.9 | Muhir-Aklil |
| *Cucurbita pepo* | mental problem | 10.0 | 8.0 | 80.0 | 0.7 | 53.3 | Qebena |
| *Hydnora johannis* | localized swelling | 15.0 | 13.0 | 86.7 | 1.0 | 86.7 | Silti |
|  | localized swelling | 20.0 | 20.0 | 100.0 | 1.0 | 100.0 | Mareqo |
| *Echinops kebericho* | general malaise (mich) | 15.0 | 10.0 | 66.7 | 1.0 | 66.7 | Cheha |
| *Eucalyptus globulus* | dengetegna | 11.0 | 7.0 | 63.6 | 0.8 | 50.0 | Muhir-Aklil |
| *Hypoestes forskaolii* | anemia | 22.0 | 21.0 | 95.5 | 1.0 | 95.5 | Muhir-Aklil |
|  | anemia | 18.0 | 9.0 | 50.0 | 1.0 | 50.0 | Silti |
|  | anemia | 26.0 | 26.0 | 100.0 | 1.0 | 100.0 | Qebena |
| *Lepidium sativum* | general malaise (mich); stabbing pain | 14.0 | 10.0 | 71.4 | 1.0 | 71.4 | Muhir-Aklil |
|  | general malaise (mich) | 10.0 | 6.0 | 60.0 | 1.0 | 60.0 | Wulbareg |
|  | general malaise (mich) | 15.0 | 14.0 | 93.3 | 1.0 | 93.3 | Mareqo |
|  | general malaise (mich) | 16.0 | 15.0 | 93.8 | 1.0 | 93.8 | Silti |
|  | general malasie (michi) | 16.0 | 11.0 | 68.8 | 1.0 | 68.8 | Cheha |
| *Ocimum lamiifolium* | general malaise (mich) | 15.0 | 9.0 | 60.0 | 1.0 | 60.0 | Sodo |
|  | general malaise(michi) | 13.0 | 11.0 | 84.6 | 1.0 | 84.6 | Wulbareg |
|  | general malaise(michi | 27.0 | 19.0 | 70.4 | 1.0 | 70.4 | Meskan |
| *Ocimum urticifolium* | general malaise (mich) | 14.0 | 10.0 | 71.4 | 1.0 | 71.4 | Wulbareg |
| *Persicaria senegalensis* | localized swelling | 9.0 | 6.0 | 66.7 | 1.0 | 66.7 | Wulbareg |
| *Prunus persica* | general malaise (mich) | 13.0 | 13.0 | 100.0 | 1.0 | 100.0 | Cheha |
| *Ruta chalepensis* | evil spirit | 18.0 | 12.0 | 66.7 | 1.0 | 66.7 | Meskan |
| *Thunbergria ruspolii* | general health (boost infant’s immunity) | 10.0 | 8.0 | 80.0 | 0.8 | 66.7 | Sodo |
| *Withania somnifera* | general malaise (mich) | 16.0 | 16.0 | 100.0 | 1.0 | 100.0 | Meskan |
|  | general malaise (mich), evil spirit | 21.0 | 19.0 | 90.5 | 1.0 | 90.5 | Silti |
|  | evil sprit | 21.0 | 19.0 | 90.5 | 1.0 | 90.5 | Cheha |
|  | evil sprit | 13.0 | 10.0 | 76.9 | 0.9 | 66.7 | Qebena |
|  | evil sprit | 15.0 | 15.0 | 100.0 | 1.0 | 100.0 | Muhir-Aklil |
|  | evil sprit | 24.0 | 20.0 | 83.3 | 1.0 | 83.3 | Mareqo |
| *Cynoglossum coeruleum* | general malaise (mich) | 11.0 | 11.0 | 100.0 | 0.8 | 78.6 | Mareqo |
| *Calpurnia aurea* | ectoparasites (fleas) | 10.0 | 10.0 | 100.0 | 0.7 | 66.7 | Qebena |
|  | ectoparasites (fleas) | 14.0 | 13.0 | 92.9 | 0.9 | 86.7 | Silti |
|  | ectoparasites (fleas) | 4.0 | 4.0 | 100.0 | 0.6 | 57.1 | Wulbareg |
| *Millettia ferruginea* | ectoparasites (fleas) | 4.0 | 4.0 | 100.0 | 0.6 | 57.1 | Wulbareg |
| *Nicotiana tabacum* | ectoparasites (leech) | 18.0 | 18.0 | 100.0 | 1.0 | 100.0 | Meskan |
|  | ectoparasites (leech) | 13.0 | 12.0 | 92.3 | 0.9 | 85.7 | Muhir-Aklil |
|  | ectoparasites (leech) | 12.0 | 11.0 | 91.7 | 0.9 | 84.6 | Cheha |
|  | ectoparasites (leech) | 21.0 | 19.0 | 90.5 | 1.0 | 90.5 | Qebena |
| *Satureja abyssinica* | abdominal parasite and bloating | 17.0 | 9.0 | 52.9 | 1.0 | 52.9 | Silti |
| *Vernonia amygdalina* | abdominal parasite and bloating | 14.0 | 8.0 | 57.1 | 1.0 | 57.1 | Sodo |
|  | abdominal parasite and bloating | 15.0 | 9.0 | 60.0 | 1.0 | 60.0 | Meskan |
| *Aloe pubescens* | wound | 10.0 | 8.0 | 80.0 | 0.8 | 66.7 | Sodo |
|  | wound | 13.0 | 8.0 | 61.5 | 0.9 | 53.3 | Silti |
|  | wound | 14.0 | 7.0 | 50.0 | 1.0 | 50.0 | Mareqo |
| *Balanites aegyptica* | eye infection | 15.0 | 12.0 | 80.0 | 1.0 | 80.0 | Mareqo |
| *Ensete ventricosum* | retained placenta | 16.0 | 9.0 | 56.3 | 1.0 | 56.3 | Meskan |
| *Fuerstia africana* | eye infection | 11.0 | 11.0 | 100.0 | 0.7 | 73.3 | Meskan |
|  | eye infection | 15.0 | 11.0 | 73.3 | 1.0 | 73.3 | Silti |
| *Persicaria senegalensis* | retained placenta | 9.0 | 5.0 | 55.6 | 1.0 | 55.6 | Wulbareg |

Ip =number of informants who use the species for a specific ailment; Iu = total number of informants who mentioned the plant for any other uses; FL (%)= Ip / Iu x 100; RPL-Relative Popularity Level ( 0 – 1); ROP- Rank Order Priority (FL* RPL).
