# Supplementary material for: Medicinal plant use practice in four ethnic communities (Gurage, Mareqo, Qebena, and Silti), south central Ethiopia
Source: J Ethnobiol Ethnomed. 2020 May 24;16:27. doi: 10.1186/s13002-020-00377-1 (PMC7245860; doi:10.1186/s13002-020-00377-1)
Supplement: Supplementary file 4 — Additional file 4. Major use categories and list of ailments/symptoms include (for compurting ICF value) [file 13002_2020_377_MOESM4_ESM.docx]

Additional file 4. Major use categories and list of ailments/symptoms include (for compurting ICF value)

| **Major use categories** | **Included ailment/symptoms (local name)** | **Major use categories** | **Included ailment/symptoms (local name)** |
| --- | --- | --- | --- |
| **Infectious and intestinal parasitic diseases (IIP)** | Diarrhea | **Diseases of the circulatory system** **(DCS)** | Hypertension |
|  | Abdominal bloating |  | Hemorrhoids |
|  | Abdominal pain or stomach cramps | **Pregnancy, childbirth and the puerperium (PCP)** | Retained placenta |
|  | Intestinal parasite, Amoebiasis |  | Lack of milk |
| **Diseases of the digestive system (DDS)** | Indigestion (Qiter) |  | Birth complications |
|  | Gastritis | **Liver complaints (LC)** | Hepatitis, Jaundice (Qoya, Seme dinku) |
|  | Flatulence | **Inflammation related to Anthrax**  **(IRA)** | Anthrax, blackleg |
| **Diseases of the respiratory system (DRS)** | Common cold | **Other unclassified (OUH)** | Epistaxis (nosebleed) (Neser) |
|  | Strong cough (Sinbabi) |  | Anemia |
|  | Asthma |  | Epilepsy (Azurit) |
|  | Pneumonia |  | Evil spirit (Lekift) |
|  | TB (Neqeresa) |  | Febrile illness (General malaise)(Michi) |
|  | Tonsillitis |  | General health (increase weight, improve strength and boost immunity for infants) |
| **Diseases of the genitourinary system (DGS)** | Gonorrhea (Emat) |  | Anorexia (loss of appetite) |
|  | Urine retention |  | Localized swelling |
| **Diseases of the musculoskeletal system (DMS)** | Lose of ability to move or paralyzed (Deme tukiy) |  | Hyperemesis (Excessive vomiting) |
|  | Rheumatic pain | **Livestock ailments** | |
|  | Stabbing pain (Wegat) | **Ectoparasites ( Livestock ailments) (ECL)** | Leech, ticks, mange mites |
|  | Back pain | **Livestock (Infectious and parasitic diseases) (LIPD)** | Antrax |
|  | Fractures |  | Blackleg |
| **Diseases of the skin and subcutaneous tissue (DSS)** | Common wart (Qintebiye, Foshe foshat) |  | Bloat |
|  | Dandruff | **Others (Livestock) (OL)** | Eye injury |
|  | Pyoderma (Koffa, Wigefye, Silensa) |  | Fattening |
|  | Wound -with boils, abscesses |  | Wound |
|  | Scabies and continuous itching |  | Swelling |
| **Diseases of the eye and adnexa** **(DEA)** | Eye infection (Wucher) |  | Retained placenta |
| **Injury, poisoning and certain other consequences of external causes (IPE)** | Skin burn |  | Rabies |
|  | Rabies |  |  |
|  | Snake bite |  |  |
|  | Herpes zoster |  |  |
| **Headache, fever and malaria (HFM)** | Fever |  |  |
|  | Headache |  |  |
|  | Malaria |  |  |
|  | Severe headache (Dan felt) |  |  |
| **Dental & oral diseases (DOD)** | Toothache |  |  |
| **Diseases of the ear and mastoid process (earache) (DEM)** | Earache |  |  |
